# Geochemical Modeling Source Provenance, Public Health Exposure, and Evaluating Potentially Harmful Elements in Groundwater: Statistical and Human Health Risk Assessment (HHRA)

**DOI:** 10.3390/ijerph19116472

**Published:** 2022-05-26

**Authors:** Abdur Rashid, Muhammad Ayub, Zahid Ullah, Asmat Ali, Seema Anjum Khattak, Liaqat Ali, Xubo Gao, Chengcheng Li, Sardar Khan, Hamed A. El-Serehy, Prashant Kaushik

**Affiliations:** 1 School of Environmental Studies, China University of Geosciences, Wuhan 430074, China; Zahid_environ225@cug.edu.cn (Z.U.); asmat@cug.edu.cn (A.A.); chengcheng@cug.edu.cn (C.L.); 2 National Centre of Excellence in Geology, University of Peshawar, Peshawar 25130, Pakistan; s_anjum@uop.edu.pk (S.A.K.); liaqat.nceg@uop.edu.pk (L.A.); 3 Department of Botany, Hazara University, Mansehra 21300, Pakistan; 54622-S19@hu.edu.pk; 4 Department of Environmental Sciences, University of Peshawar, Peshawar 25120, Pakistan; sardar@uop.edu.pk; 5 Department of Zoology, College of Science, King Saud University, Riyadh 11451, Saudi Arabia; helserehy@ksu.edu.sa; 6 Instituto de Conservación y Mejora de la Agrodiversidad Valenciana, Universitat Politècnica de València, 46022 Valencia, Spain; prakau@doctor.upv.es

**Keywords:** groundwater contamination, carcinogenic/noncarcinogenic risk, spatial distribution, geochemical speciation, mineral phases

## Abstract

Groundwater contamination by potentially harmful elements (PHEs) originating from the weathering of granitic and gneissic rock dissolution poses a public health concern worldwide. This study investigated physicochemical variables and PHEs in the groundwater system and mine water of the Adenzai flood plain region, in Pakistan, emphasizing the fate distribution, source provenance, chemical speciation, and health hazard using the human health risk assessment HHRA-model. The average concentrations of the PHEs, viz., Ni, Mn, Cr, Cu, Cd, Pb, Co, Fe, and Zn 0.23, were 0.27, 0.07, 0.30, 0.07, 0.06, 0.08, 0.68, and 0.23 mg/L, respectively. The average values of chemical species in the groundwater system, viz., H^+^, OH^−^, Ni^2+^, Mn^2+^, Mn^3+^, Cr^3+^, Cr^6+^, Cu^+^, Cu^2+^, Cd^2+^, Pb^2+^, Pb^4+^, Co^2+^, Co^3+^, Fe^2+^, Fe^3+^, and Zn^2+^, were 1.0 × 10^−4^ ± 1.0 × 10^−6^, 1.0 × 10^−4^ ± 9.0 × 10^−7^, 2.0 × 10^−1^ ± 1.0 × 10^−3^, 3.0 × 10^−1^ ± 1.0 × 10^−3^, 1.0 × 10^−22^ ± 1.0 × 10^−23^, 4.0 × 10^−6^ ± 2.0 × 10^−6^, 4.0 × 10^−11^ ± 2.0 × 10^−11^, 9.0 × 10^−3^ ± 1.0 × 10^−2^, 2.0 × 10^−1^ ± 2.0 × 10^−3^, 7.0 × 10^−2^ ± 6.0 × 10^−2^, 5.0 × 10^−2^ ± 5.0 × 10^−2^, 2.0 × 10^−2^ ± 1.5 × 10^−2^, 6.0 × 10^−2^ ± 4.0 × 10^−2^, 8.0 × 10^−31^ ± 6.0 × 10^−31^, 3.0 × 10^−1^ ± 2.0 × 10^−4^, 4.0 × 10^−10^ ± 3.0 × 10^−10^, and 2.0 × 10^−1^ ± 1.0 × 10^−1^. The mineral compositions of PHEs, viz. Ni, were bunsenite, Ni(OH)_2_, and trevorite; Mn viz., birnessite, bixbyite, hausmannite, manganite, manganosite, pyrolusite, and todorokite; Cr viz., chromite and eskolaite; Cu viz., CuCr_2_O_4_, cuprite, delafossite, ferrite-Cu, and tenorite; Cd viz., monteponite; Pb viz, crocoite, litharge, massicot, minium, plattnerite, Co viz., spinel-Co; Fe viz., goethite, hematite, magnetite, wustite, and ferrite-Zn; and Zn viz., zincite, and ZnCr_2_O_4_ demarcated undersaturation and supersaturation. However, EC, Ca^2+^, K^+^, Na^+^, HCO_3_^−^, Cr, Cd, Pb, Co, and Fe had exceeded the WHO guideline. The Nemerow’s pollution index (NPI) showed that EC, Ca^2+^, K^+^, Na^+^, HCO_3_^−^, Mn, Cd, Pb, Co, and Fe had worse water quality. Principal component analysis multilinear regression (PCAMLR) and cluster analysis (CA) revealed that 75% of the groundwater contamination originated from geogenic inputs and 18% mixed geogenic-anthropogenic and 7% anthropogenic sources. The HHRA-model suggested potential non-carcinogenic risks, except for Fe, and substantial carcinogenic risks for evaluated PHEs. The women and infants are extremely exposed to PHEs hazards. The non-carcinogenic and carcinogenic risks in children, males, and females had exceeded their desired level. The HHRA values of PHEs exhibited the following increasing pattern: Co > Cu > Mn > Zn > Fe, and Cd > Pb > Ni > Cr. The higher THI values of PHEs in children and adults suggested that the groundwater consumption in the entire region is unfit for drinking, domestic, and agricultural purposes. Thus, all groundwater sources need immediate remedial measures to secure health safety and public health concerns.

## 1. Introduction

Groundwater is a vital freshwater resource for humans worldwide [1,2,3]. Most groundwater is used in domestic, industrial, and agricultural sectors [4,5]. Groundwater is composed of inorganic substances that contain potentially harmful elements (PHEs) that contaminate groundwater worldwide [6]. The quality of groundwater has deteriorated with the growth of industrialization, urbanization, and mining activities [7,8,9]. The presence of abundant mines and improper management mainly causes severe environmental impacts on the groundwater system of Adenzai mining and the flood plain region of Pakistan. Moreover, the evaluation of groundwater resources that measures quality and availability for domestic and commercial suitability is important [10,11]. Apart from abundant mines, waste disposal, and industrial effluents, the concentrations of the groundwater PHEs in the surrounding water aquifer increases with seasonal variability due to precipitation, and it gradually reduces as a result of evaporation processes [12,13,14,15]. The seasonal distribution of PHEs during precipitation and evaporation is evident due to the alteration of the groundwater tables from wet to dry showing seasonal fluctuations [16,17,18,19]. However, the level of the groundwater table varies from place to place, even within the same location. Changes in precipitation across seasons and years produce fluctuations in the water table level. The water table rises in late winter and early spring when snow melts and there is abundant precipitation [20]. While groundwater levels tend to be low in the summer and autumn seasons due to evaporation and dryness, they are abundant during the rainy season or in the winter and spring [10,21].

Most of the recent literature reports that the term heavy elements has gradually interchanged and shifted to potentially harmful elements (PHEs) [6]. Groundwater sources contain potentially toxic elements, which includes poisonous elements [6,22,23]. The current study includes the following potential PHEs: nickel (Ni), manganese (Mn), chromium (Cr), copper (Cu), cadmium (Cd), lead (Pb), cobalt (Co), iron (Fe), and zinc (Zn). Though the higher levels of PHEs in the groundwater are toxic in nature, a few of them are used as food supplements [22,23]. Therefore, groundwater contamination is of prime importance, particularly potentially harmful elements (PHEs) [6,24]. Moreover, the higher content of PHEs in groundwater aquifers is extremely hazardous and causes health implications.

The human health risk assessment model (HHRA-model) is a helpful technique for understanding the severity of PHEs posed through groundwater ingestion [25]. According to the world health organization, about 80% of waterborne diseases are caused by water pollution [26,27,28]. Preferably, water pollution is a significant health concern in different societies and agricultural zones worldwide [29]. The excessive ingestion of groundwater containing PHEs such as Cd, Cr, Cu, Mn, Co, Ni, Pbs, and Zn causes potential ecological and biological health risks [7,24,30,31,32]. The contamination of groundwater resources has important repercussions for human health. PHEs are highly harmful due to their bioavailability, harmfulness, and persistency, which ultimately causes water-borne diseases [33,34,35].

The study of PHEs within the groundwater aquifer is challenging and requires evaluation of a vast data set and modern methodologies. However, the identification of PHEs in the groundwater system needs several evaluation methods. These evaluation methods include factor analysis (FA), water quality grading (WQG), the Nemerow pollution index (NPI), the pollution load index (PLI), principal component analysis multilinear regression (PCAMLR), cluster analysis (CA), agriculture indexing, the Fuzzy comprehensive method (FCM), GIS interpolation, quantile-quantile Q-Q plot, saturation indexing, and groundwater quality indexing (GWQI). These methodologies include multivariate statistical analysis that sheds light on their basic information regarding similarity and dissimilarity in comparing groundwater results. These statistical approaches describe the controlling role of various hydrogeochemical processes that govern the hydrochemistry of PHEs in surrounding water aquifers [13,30,36,37]. All these evaluation methods have deep influences on the enrichment of PHEs in the water system. The PCAMLR and CA determine the percent contribution of pollution sources, while GIS and Q-Q plots reveal the spatial distribution of the water pollutant. However, GWQI, WQG, and NPI assess the groundwater quality status by distributing water into different groups. Thus, environmental scientists and water chemists can easily differentiate between natural and anthropogenic groundwater contaminants [29,38]. Thus, a large data set is accomplished instead of losing critical information; therefore, accurate hydrogeochemical observations of the studied aquifers are obtained.

Moreover, PHEs are released into the groundwater system from natural and anthropogenic sources. The predominant geogenic source of PHEs accounted, viz., weathering of rocks, soil erosion, volcanic activities, and mineral ore deposits [39]. At the same time, the anthropogenic sources include mining, agriculture practices, and industrial wastewater discharges [30,40,41,42]. Mostly, humans are severely affected by industrial discharges, which ultimately make their way to natural water bodies [43]. The high content of PHEs in drinking groundwater in the aquatic system is a significant concern due to its adverse impact on humans, plants, animals, and the ecosystem [44]. The governments and associated organizations in developing countries, including Pakistan, are trying to ensure safe provisions for drinking groundwater. Most people in developing nations have no access to safe groundwater [45]. Water contamination is primarily due to intermittent water supply, insufficient chlorination, and sewage systems [46]. Worldwide, the people of most developing nations suffer from either chronic or acute water-borne diseases. The higher population growth and impoverished socioeconomic development, scarce water resources, and poor sanitation processes can lead to worse living conditions [47]. Scientists claim that women play an essential role in water sanitation, health, management, and the conservation of water resources.

In the above discussion, we studied the groundwater contamination with potentially harmful elements (PHEs), fate distribution, source provenance, geochemical speciation, and health risk exposure assessment in the Adenzai flood plain region of Pakistan. Moreover, in Pakistan groundwater, contamination problems significantly linked to urbanization, industrialization, agriculture practices, mining actions, and groundwater uptake from the shallow aquifer [40,48]. To our knowledge, this is the first systematic study to evaluate the public health hazard that is posed due to PHE contamination in the groundwater system around abundant mines and the flood plain region of Adenzai, in northern Pakistan. Moreover, the fine dust particles emitted from mining activities degrade the surrounding environment, including the groundwater system. Several mines have existed, and their water has been extensively used for drinking, domestic, and agriculture purposes [9]. However, the depth profile of PHE’s contamination in the groundwater and their associated health risks have not been explored in this area. Therefore, considering the population, public health, geology, and anthropogenic inputs, this study aims to: (1) investigate the depth profile of potentially harmful element (PHE) contamination levels in the drinking groundwater; (2) evaluate the human health hazards, viz., noncarcinogenic and carcinogenic, due to the ingestion of contaminated groundwater by using human health risk assessment (HHRA-model); and (3) understand the geogenic and anthropogenic origin of PHE contamination in the groundwater using principal component analysis multilinear regression (PCAMLR). Moreover, the question survey was designed for both genders, including children and adults, to collect information related to health, economic status, and groundwater consumption rate. The findings of this study are helpful to the residents in general, while they are specific for the government to take safety measures, safeguard public health, and create awareness about PHEs in the entire community.

## 2. Materials and Methods

### 2.1. Study Area Description

**Figure 1:**
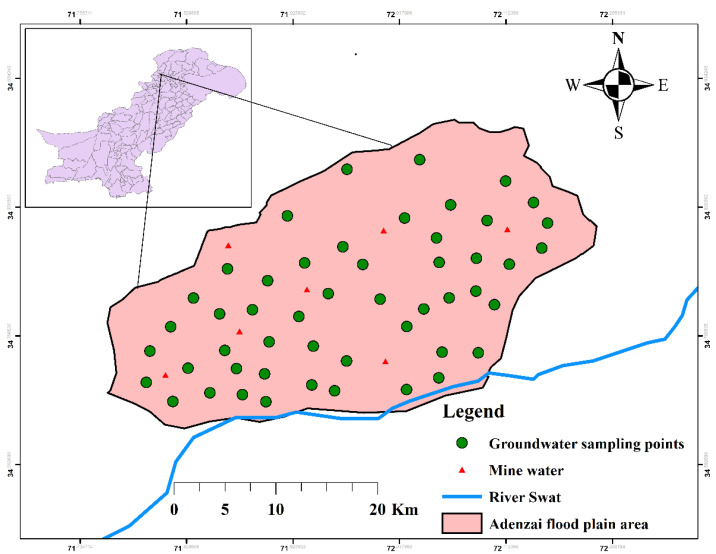
Representation of the location map outline groundwater sampling points in the study area.

The Adenzai flood plain region in northern Pakistan is located between 34°–39° Northing and 71°–72° Easting (Figure 1). It occupies an area of 140 km^2^ and has a total population of 130,000 individuals. Topographically, the study area has been occupied by the foothills of Hindukush ranges in the northeast to the southwest zone [49]. Geographically, the site is bounded with district Upper Dir in the north, district Swat in the east, district Malakand in the south, and Bajaur Agency in the west. The groundwater was collected from three hydrological environments: shallow depth, mid-depth, and deeper depth. The groundwater has been extensively utilized for drinking, domestic, and agriculture practices. The people of this region mostly use groundwater extracted from springs, hand pumps, dug wells, tube wells, municipal communities, and shallow mines. River Swat and Panjkora are flowing at the front and backside of the study area.

### 2.2. Climate, Hydrology, and Hydrogeology

**Figure 2:**
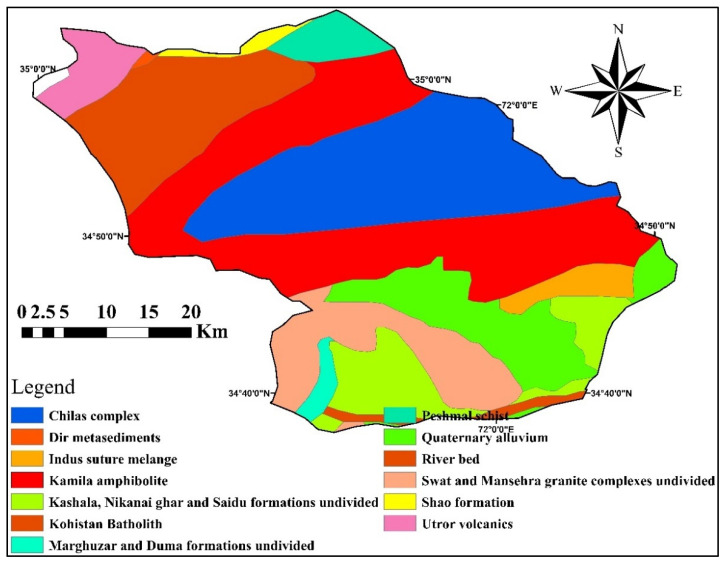
The identified geological formations in the Adenzai flood plain region of Pakistan.

The area’s hydrology defines how much groundwater is utilized by the local residents for drinking and domestic purposes. The residents consume from different groundwater resources, including hand pumps, springs, tube wells, and dug wells. These groundwater resources receive surface recharge from River Swat and Panjkora and snowfall on the surrounding mountains. The water that has lesser depth usually has a lower temperature and is predominantly used for domestic, industrial, and agricultural purposes. The area’s hydrogeology includes rock minerals such as epidote, plagioclase, quartz, galena, hornblende, apatite, and sphene; these rocks interact with the groundwater [50]. Chackdara (Adenzai), granite gneisses rock, covers 60 km^2^ in the north of the Malakand granitic zone. The geological setting (outlined geological compositions containing Chackdara orthogenesis) is a type of intrusive igneous rock usually called Mekhband, and formations extensively prevail in the area (see Figure 2). Most rock formations exist as coarse-granite, intermediate, and uniform; all these rocks include essential minerals of quartz, muscovite, biotite plagioclase, magnetite, and feldspar granite gneissic gem.

### 2.3. Preparation and Analysis of Groundwater Samples

The groundwater samples were collected from the Adenzai flood plain region, northern Pakistan, for physiochemical and PHE analysis. In the study, the groundwater samples were filtered using 0.42-µm Whatman filter paper to safeguard sophisticated instruments [51]. The pH of groundwater samples was adjusted with the addition of 1–2 mL of ultrapure HNO_3_; afterward, the samples were kept in a refrigerator at 4 °C until further analysis [29,52].

The groundwater samples (*n* = 50) were collected from shallow groundwater (*n* = 24), mid-depth groundwater (*n* = 14), deeper depth groundwater (*n* = 12), and mine waters (*n* = 7). The samples were collected from different sources, including bore wells, springs, open dug wells, hand pumps, and community wells. Before collecting the samples, a handheld GPS (HC Garmin) was used to identify the geographic coordinates of each sampling point (see Figure 1). The samples were stored in 100-mL polyethylene bottles, rinsed and soaked three times with double deionized water. All the water samples were gathered for the cations, and PHEs analysis was acidified by putting in 2–3 drops of HNO_3_. The addition of acid in water samples avoided the precipitation of PHEs, and most importantly, it protected the activities of microbes in the groundwater. All groundwater samples were analyzed three times, and the mean was included in the results. The instrument used to determine PHEs was Inductively Coupled Plasma Mass Spectrometry “ICPMS” (Agilent 7500 ICPMS model manufactured by the USA), under standardized operating conditions. The quantization limits for Ni, Cr, Cu, Cd, Pb, and Zn were recorded to be 0.5, 0.1, 0.1, 0.02, 0.5, and 0.5 mg/L, respectively. The percentile recovery of PHEs, viz., Ni, Mn, Cr, Cu, Cd, Pb, Co, Fe, and Zn, were recorded to be 96%, 97%, 95%, 97%, 95%, 93%, 91%, 98%, and 95%, respectively.

### 2.4. Questioner Survey

To find the overall adverse impacts of contaminated groundwater, a question-based interview was conducted in the Adenzai region, northern Pakistan. The medical and environmental experts randomly surveyed 1500 households out of 130 thousand individuals, approximately, and this was the public help that was involved in this research. The local respondents included males and females with a secondary level of education. The age of children ranged from 10–14 and adults 15–65 years.

The questionnaire survey included age, body weight, monthly income, education, waterborne diseases, bathing habits, migration habits, smoking or non-smoking habits, occupational exposure, and other health-associated problems. The questionnaires were filled out by respondents of the proposed area with great care regarding information. The expert of the medical team helped the respondents in understanding the questionnaire, and sometimes the questions were explained verbally in their native language. The comments section was included in the designed questionnaire to encourage the respondents to give feedback and add some new comments/information regarding the need for the survey. The survey team explained the queries for the local consumers, who consume from different groundwater resources such as dug wells, tube well, springs, hand pumps, community wells, and mine water. Moreover, meetings and interviews were arranged with the expert doctors of the local hospitals and health units who work to collect waterborne diseases in the proposed study area.

### 2.5. Health Risk Assessment

The purpose of the risk assessment included chronic daily ingestion (CDI), hazard quotient (HQ), and health indices (HI) to calculate the health risk via exposure to PHE concentrations through groundwater ingestion. The impacts of this risk assessment are expressed in terms of carcinogen and non-carcinogen using the HHRA model [53]. The exposure pathways included oral ingestion, dermal, and inhalation. However, oral ingestion is the most important route for PHE ingestion in the ground and surface water, soil, and food [6,24]. Therefore, oral ingestion was considered for carcinogenic and noncarcinogenic exposure in this study.

The noncarcinogenic and carcinogenic exposures of PHEs were determined using the following mathematical equations. According to Chrostowski and USEPA, the CDI via water ingestion can be calculated using modified Equation (1):

CDI = CW × R × EF × ED/BW × AT(1)

where CW, IR, EF, ED, BW, and AT represent the PHE concentrations in groundwater (mg/L), ingestion rate of groundwater, exposure frequency (365 days year^−1^), exposure duration (30 years), body weight, and average time 365 days year^−1^ × ED for ‘non-carcinogenic and 365 days/year × 70 years for ‘carcinogens’ [44,54,55,56]. After CDI calculation, we determined the HQ for noncarcinogenic risk using Equation (2). The USEPA database arranged the (RfD), oral reference dose standards, for groundwater PHEs, including Cd (5.0 × 10^−4^), Cr (1.5), Cu (3.7 × 10^−2^), Mn (1.4 × 10^−1^), Ni (2.0 × 10^−2^), Pb (3.6 × 10^−2^), Zn (3.0 × 10^−1^) mg/kg-day^−1^, respectively [56]. The exposed populations were assumed to be safe when the value of HQ < 1 [54].

(2) HQ=CDI/RfD

However, the carcinogenic risk level of PHEs was determined by Equation (3):

CR = CDI × CSF(3)

The CR is the cancer risk, and ‘CSF’ is the cancer slope factor measured in mg/kg-day^−1^ [54].

### 2.6. Statistical Analysis

All the groundwater data, including descriptive statistics such as range, mean, standard deviation, and Pearson correlation with a significance level of 0.05 level, were employed and calculated using the XLSTAT 2021 computer package. The multivariate statistical analysis included the pollution index (PI), cluster analysis (CA), and principal component analysis multilinear regression (PCA-MLR), which were performed by mixing the technique via SPSS, IBM statistic software version 20, and XLSTAT 2021 [31,38,43].

### 2.7. Pollution Index

Pollution indices (PI) are considered a useful statistical technique used to define the contribution and impacts of different groundwater contaminants. However, PI for groundwater is calculated by using Equation (4):

(4) $$\mathrm{PI}=\overset{n}{\underset{i=1}{\sum }}\mathrm{Ci}–\mathrm{Si}/\mathrm{Si}$$

### 2.8. Nemerow’s Pollution Indexing (NPI)

Nemerow’s pollution indexing (NPI) is an important method used to test the quality of the groundwater. This testing approach is also known as Row’s pollution index (RPI). The steps for calculating NPI index values are very simple when compared to other water quality assessment methods. This method was used to check the water quality indexing of groundwater sources compared to mine water for nineteen parameters. Mathematically, the following formula was used to calculate the NPI [57].

(5) $$\mathrm{Nemerow}’s\;\mathrm{pollution}\;\mathrm{Index}=\frac{C_{n}}{S_{n}}$$

where C_n_ is the concentration of the nth parameter of groundwater, and S_n_ is the standard limit of the nth parameter.

### 2.9. Cluster Analysis

Clustering is a multivariate method used to assemble the groundwater data and to form different groups. Cluster analysis classifies groundwater variables and formulates groups based on homogeneity and heterogeneity; the variables of most similar samples fall within the same groups. Those dissimilar in characteristics developed different clusters. The most useful cluster method exhibited as hierarchical agglomerative clustering provides a homogenous relationship for the overall data set and is represented by a dendrogram plot [58]. The dendrogram typically provides three groups: cluster 1, cluster 2, and cluster 3. Similarly, the Euclidean distance was measured and represented invariance, which resembles two groundwater samples [59]. Clustering analysis was calculated via Ward’s method, which evaluated the distance among clusters to minimize the square sum of two sets formed during analysis [60,61].

### 2.10. Principal Component Analysis Multilinear Regression

Principal component analysis multilinear regression (PCA-MLR) is a valuable technique used to understand the pollution sources of PHEs in groundwater in terms of % age contribution. The pollution index (PI) was calculated for overall groundwater parameters [9]. Then, PCA was measured through the Varimax rotation reduction dimension method [13,29]. The three components, F1, F2, and F3, and pollution load (PI) were loaded in the spreadsheet of SPSS (version 20). The PI was loaded as a dependent value, and the three factors were loaded as independent values after regression. The R^2^ values were taken from the model summary, then the R^2^ of individual factors F1, F2, and F3 were calculated by removing one component and leaving all the other components as independent values. After the R^2^ calculation, the R^2^ difference was estimated by subtracting the R^2^ value of each element from the overall R^2^ values. The percentage contribution was calculated by summing the R^2^ differences.

### 2.11. Mapping

A global positioning system (GPS name HC Garmin) handheld instrument was used to collect the geographic coordinates of each sample point. The coordinates were designed in an excel sheet and plotted in ArcGIS software version 10.6 to generate a study area map (Figure 1) and geological map (Figure 2). The distribution map was designed and described the pattern of groundwater contaminants in the form of low and high concentrations to understand the contamination level of PHEs in the groundwater aquifer.

### 2.12. Quality Assurance and Quality Control

The precision and accuracy of the PHEs in the groundwater analysis were ensured by using certified reference materials (CRMs). The reference materials were obtained from the Ministry of Environmental Protection Agency (Beijing, China) and were used for groundwater PHEs analysis. The certified reference materials used for groundwater were GSB 07-1183-2000, GSBZ 50004-88, GSB 07-1185-2000, and GSBZ 50016-90. Different standards and reagent blanks were also used to ensure accurate and precise results. Moreover, the chemical ion balance error (CIBE) was calculated in meq/L [29,62]; to check the accuracy of the water found to be ±5% errors, Equation (6) was used.

(6) $$\mathrm{ICBE}=\frac{\sum \mathrm{Cations}\;-\sum \mathrm{Anions}}{\sum \mathrm{Cations}\;+\sum \mathrm{Anions}}\times 100\;$$

## 3. Results and Discussion

### 3.1. Geochemical Profile of Physicochemical Variables and Potential Harmful Elements

Table 1 represents the range and mean values of PHEs and the associated physicochemical groundwater and mining water variables in the Adenzai flood plain region of Pakistan. Groundwater and mine water findings revealed that most PHEs had exceeded the WHO guidelines [63]. The groundwater samples (*n* = 50) were grouped into three classes based on their depth, viz., shallow (depth ≤ 40 m), mid-depth (41–80 m), and deeper depth (>80 m), respectively. The groundwater results based on their depth and mine profile altered their geochemical composition. The ranges of concentrations of pH, EC, temp, depth, and TDS in the shallow depth groundwater were 7.2–8.3, 212–1288 µS/cm, 24.5–26.6 °C, 25.0–40.0 m, and 210–800 mg/L, respectively. The pH values of groundwater samples were slightly alkaline and were within the acceptable limit of the World Health Organization [63]. However, the ranges of concentrations of the cations, viz., Ca^2+^, Mg^2+^, K^+^, and Na^+^, in the shallow groundwater were 27–100, 15.0–33.0, 4.5–18.9, and 55.0–350.0 mg/L, respectively, whereas the ranges of concentrations of the major anions, viz., HCO_3_, Cl, and SO_4_, were 210–850, 80–150, and 115–241 mg/L, respectively. The ranges of concentrations of PHEs in shallower groundwater were 0.05–0.53, 0.06–0.50, 0.03–0.15, 0.03–1.90, 0.01–0.31, 0.01–0.20, 0.23–1.34, and 0.11–0.65 mg/L, respectively. The increasing trend of PHEs observed in the shallow groundwater followed Cu > Fe > Zn > Ni > Mn > Cd > Co > Pb > Cr, respectively.

The ranges of concentrations of pH, EC, temp, depth, and TDS in mid-depth groundwater were 7.0–8.1, 333–1030 µS/cm, 24.6–27.2 °C, 41.0–80.0 m, and 210–635 mg/L, respectively. Similarly, the ranges of values of the cations, viz., Ca^2+^, Mg^2+^, K^+^, and Na^+^ in the mid-depth groundwater were 34.0–85.0, 18.0–37.0, 4.5–10.8, and 45.0–170.0 mg/L, and the ranges of the anions, viz., HCO_3_, Cl, and SO_4_, were 180–335, 55.0–145.0, and 89.3–236.3 mg/L, respectively. The ranges of concentrations of PHEs in mid-depth groundwater were 0.04–0.54, 0.08–0.50, 0.03–0.17, 0.03–0.45, 0.01–0.23, 0.01–0.16, 0.03–0.23, 0.24–1.56, and 0.12–0.37 mg/L, respectively. The increasing trend of PHEs recorded in the mid-depth groundwater assumed Fe > Ni > Mn > Cu > Zn > Cd > Co > Cr > Pb, respectively.

The ranges of concentrations of pH, EC, temp, depth, and TDS in deep groundwater were 7.2–8.1, 469–1121 µS/cm, 24.5–26.2 °C, 85.0–115.0 m, and 300–680 mg/L, respectively. Likewise, the ranges of concentrations of the cations, viz., Ca, Mg, K, and Na, in the deeper groundwater were 28.0–120.0, 18.0–45.0, 0.9–10.8, and 22.0–150.0 mg/L, and the ranges of the anions, viz., HCO_3_, Cl, and SO_4_, were 190.0–330.0, 80.0–135.0, and 78.8–152.3 mg/L, respectively. The ranges of concentrations of PHEs in deeper groundwater were 0.03–0.40, 0.08–0.35, 0.01–0.08, 0.01–0.24, 0.01–0.06, 0.01–0.04, 0.03–0.21, 0.11–0.31, and 0.12–0.35 mg/L, respectively. The increasing trend of PHEs in the deep groundwater was Ni > Mn > Zn > Fe > Cu > Co > Cr > Cd > Pb, respectively.

The groundwater results of this study were compared with Singh et al., 2018; Jehan et al., 2019; Agusa et al.; and de Joode et al., 2016. The results declared higher concentrations of PHEs and physicochemical variables than those reported by the researchers above [52,64,65,66]. However, the PHE results of the current studies are consistent with those outlined by [6]. It was observed that Cr has excellent mobility under oxidizing conditions. The highest Ni, Mn, and Cr concentrations were observed in the mid-depth groundwater samples attributed to the weathering and dissolution of ultramafic rock, containing minerals such as dunite and peridotite in the bed host chromite deposits.

The increasing geographic trend indicated the following pattern, according to the depth profile of groundwater: shallow groundwater > mid-depth groundwater > deep groundwater, respectively (see Table 1). Overall, 48%, 30%, 64%, 62%, and 62% of the groundwater samples contribute to Cr, Cd, Pb, Co, and Fe contamination in the entire study area. The Cd contamination in groundwater was mainly released from agriculture practices, sewage sludge, and corrosion of plumbing, water pipe, and phosphate fertilizers [44]. At the same time, the highest Pb concentrations up to 0.20 mg/L were reported in the shallow groundwater in the Badwan area. This study showed that the groundwater system is heavily contaminated with Pb due to leaching and transportation from the plumbing system [63]. However, the Co and Fe content in the groundwater of the Adenzai area exceeded the WHO guideline values of 0.04 and 0.3 mg/L. Therefore, the groundwater was unfit for drinking and domestic purposes regarding Cr, Cd, Pb, Co, and Fe. However, the Ni, Mn, Cu, and Zn concentrations showed no contamination in the study area. Overall, the groundwater samples exceeded the WHO guidelines for EC, Ca, K, Na, HCO_3_, and PHEs, viz., Cr, Cd, Pb, Co, and Fe, respectively. However, their percentage exceedance was 90%, 4%, 2%, 12%, 10%, 46%, 32%, 62%, 56%, and 62%, respectively, whereas the pH, TDS, Mg, Cl, SO_4_, Ni, Mn, Cu, and Zn concentrations in the groundwater system were within the Pak, EPA, and WHO guideline values [63,67].

### 3.2. Geochemistry of Underground Mines Water

The existence of mines and mineral deposits in the flood plain contributes to PHEs in the groundwater system. Mainly, PHEs occur in solid phases with a moderate tendency of mobilization in the groundwater system; though, it can be released into surrounding aquifers under some conditions. Table 1 describes the geochemical profile of mine water. The ranges of concentrations of pH, EC, temp, depth, and TDS in the mine’s water were 7.6–8.2, 1650–1850 µS/cm, 24.1–27.8 °C, 24.0–35.0 m, and 1050–1280 mg/L, respectively. Likewise, the ranges of values of the cations, viz., Ca^2+^, Mg^2+^, K^+^, and Na^+^, in the deeper groundwater were 20.0–29.0, 10.0–21.0, 1.8–12.0, 335–410 mg/L, and the ranges of the anions, viz., HCO_3_^−^, Cl^−^, and SO_4_^2−^, were 610–680, 80–120, and 325–350 mg/L, respectively. The ranges of concentrations of PHEs in mine water were 0.32–0.52, 0.65–1.58, 0.65–1.58, 1.20–2.25, 0.35–0.48, 0.25–0.58, 0.24–0.52, 2.10–2.85, and 0.45–0.90 mg/L, respectively.

Groundwater pumped from the underground mine situated in the mafic and ultramafic rock showed elevated concentrations of PHEs compared to the background water. It was observed that the local inhabitants of this area used mined water for drinking and domestic purposes. Mainly, neutral to alkaline pH levels and elevated concentrations of PHEs were observed in the underground mining water. The author observed that the local residents consume mine water for their domestic use. The PHE analysis of mine water showed that Mn, Cr, Pb, Cd, Co, and Fe had exceeded the WHO guideline values, while the rest of the PHEs showed satisfactory results. The increasing trend of PHEs recorded in the mine water was Fe > Mn > Zn > Pb > Ni > Co > Cd > Cr > Cu, respectively. The PHEs, viz., Pb, Cd, Cr, Co, and Mn, suggest that these metals are highly harmful from a health perspective. The elevated levels of PHEs in the underground mine water of the study area should not be used for drinking, domestic, or agriculture purposes.

### 3.3. Geochemical Facies and Control Mechanism

**Figure 3:**
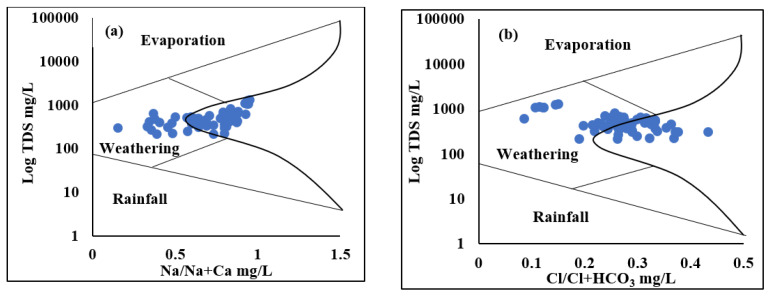
The thematic plot represents the major ion composition controlling the groundwater profile of the Adenzai flood plain region: (**a**) Na^+^/Na^+^ + Ca^++^ mg/L against Log TDS; (**b**) Cl^–^/Cl^–^ + HCO_3_^–^ mg/L against Log TDS.

Gibb’s plot and the Chadha diagram were used to understand the hydrogeochemical regime and geochemical composition of groundwater samples in the Adenzai flood plain area. The Chadha diagram is the expanded version of the piper trilinear diagram demonstrating that most of the water samples were mixed cations, with HCO_3_^−^ and Cl^−^ as the dominating ions, forming NaHCO_3_ and NaCl type water. According to Gibb’s plot, the geochemistry of the groundwater samples was mainly controlled by weathering and recharge mechanisms (see Figure 3). At the same time, precipitation and evaporation were the least significant in the entire area.

**Figure 4:**
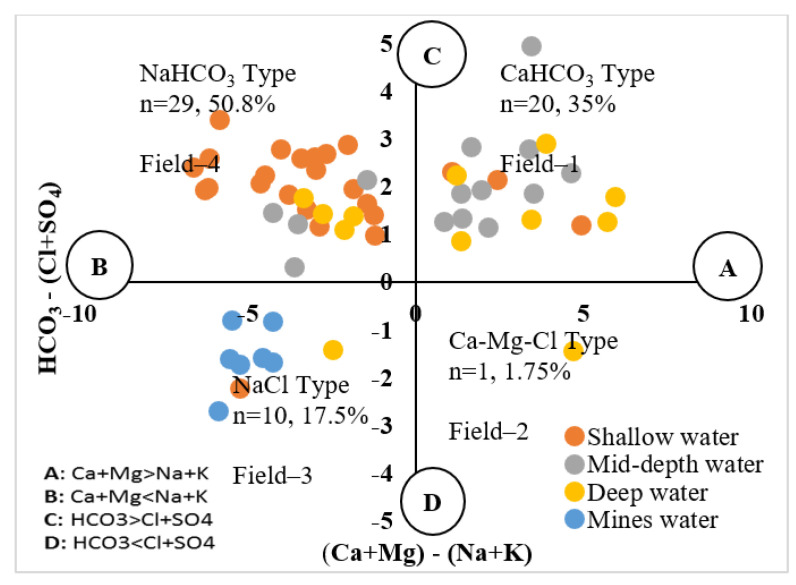
Chadha scatter diagram that indicates the composition of groundwater type in the Adenzai region. Field-1 indicates Ca-HCO_3_-type water compiling weathering and a recharge mechanism, and Field-2 shows that Ca-MgCl groundwater determines the ionic reverse exchange. Field-3 reveals evaporation forming the NaCl type, and Field-4 defines NaHCO_3_-type elaborate ion-exchange processes.

However, Chadha and Tamta designed a new hydrogeochemical sequence for groundwater classification using a difference of cations (Ca^2+^ + Mg^2+^) and (Na^+^ + K^+^) and anions such as HCO_3_^–^ and (Cl^–^ + SO_4_^2−^) as concentrations calculated in meq/L as a percentile [68]. The groundwater samples of the entire study area showed four water types. The primary groundwater composition was based on NaHCO_3_ and accounted for 50.8% contribution, 35% CaHCO_3_ type, NaCl type 17.5%, and Ca-Mg-Cl contributed 1.75%, respectively (see Figure 4). The geochemical facies of shallow groundwater were mainly controlled by NaHCO_3_ type, while mid-depth and deep groundwater were controlled by CaHCO_3_ type, and regardless of the type of mine water, it was occupied by NaCl type. The main factors that influenced the shallow groundwater were ion exchange processes and the alteration of CaHCO_3_ type into NaHCO_3_ type, whereas weathering and recharge mechanisms mainly controlled mid-depth and deep groundwater, which is composed of CaHCO_3_ type. At the same time, mine water is controlled by evaporation processes that play significant inputs in the formation of saline NaCl-type water, whereas, Ca-Mg-Cl-type water is formed due to the reverse ion-exchange method. The control mechanism using Gibb’s plot and the geochemical results of the current study were compared with Jehan et al., 2020, and the findings were consistent [52].

### 3.4. Geochemical Speciation of PHEs

Table 2 reveals the geochemical speciation of groundwater and mine water in the Adenzai flood plain region. The ranges of concentrations of H^+^, OH^−^, Ni^2+^, Mn^2+^, Mn^3+^, Cr^3+^, Cr^6+^, Cu^+^, Cu^2+^, Cd^2+^, Pb^2+^, Pb^4+^, Co^2+^, Co^3+^, Fe^2+^, Fe^3+^, and Zn^2+^ were recorded in the groundwater of the Adenzai flood plain region up to 1.0 × 10^−4^–1.15 × 10^−4^, 1.0 × 10^−4^–1.05 × 10^−4^, 3.0 × 10^−2^–5.0 × 10^−1^, 6.0 × 10^−2^–5.0 × 10^−1^, 2.0 × 10^−23^–1.94 × 10^−22^, 7.0 × 10^−7^–7.12 × 10^−6^, 8.0 × 10^−12^–8.06 × 10^−11^, 3.0 × 10^−4^–5.63 × 10^−2^, 7.0 × 10^−3^–1.30 × 10^−2^, 1.0 × 10^−2^–1.0 × 10^−1^, 8.0 × 10^−3^–2.0 × 10^−2^, 1.0 × 10^−2^–1.0 × 10^−1^, 7.0 × 10^−3^–2.0 × 10^−2^, 9.0 × 10^−32^–2.3 × 10^−30^, 6.0 × 10^−2^–2.0 × 10^−1^, 7.0 × 10^−11^–1.05 × 10^−9^, and 6.0 × 10^−2^–2.0 × 10^−1^, respectively. Similarly, the mean ± SD values for studied groundwater variables were 1.0 × 10^−4^ ± 1.0 × 10^−6^, 1.0 × 10^−4^ ± 9.0 × 10^−7^, 2.0 × 10^−1^ ± 1.0 × 10^−2^, 3.0 × 10^−1^ ± 1.0 × 10^−2^, 1.0 × 10^−22^ ± 5.0 × 10^−23^, 4.0 × 10^−6^ ± 2.0 × 10^−8^, 4.0 × 10^−11^ ± 2.0 × 10^−10^, 9.0 × 10^−3^ ± 1.0 × 10^−2^, 2.0 × 10^−1^ ± 2.0 × 10^−3^, 7.0 × 10^−2^ ± 6.0 × 10^−3^, 5.0 × 10^−2^ ± 1.0 × 10^−2^, 2.0 × 10^−1^ ± 2.0 × 10^−3^, 6.0 × 10^−2^ ± 4.0 × 10^−2^, 8.0 × 10^−31^ ± 6.0 × 10^−31^, 3.0 × 10^−1^ ± 2.0 × 10^−3^, 4.0 × 10^−10^ ± 3.0 × 10^−10^, and 2.0 × 10^−1^ ± 1.0 × 10^−1^, respectively.

The ranges of concentrations of H^+^, OH^−^, Ni^2+^, Mn^2+^, Mn^3+^, Cr^3+^, Cr^6+^, Cu^+^, Cu^2+^, Cd^2+^, Pb^2+^, Pb^4+^, Co^2+^, Co^3+^, Fe^2+^, Fe^3+^, and Zn^2+^ observed in the mine water in the Adenzai flood plain region were 1.0 × 10^−4^–1.0 × 10^−4^, 1.0 × 10^−4^–1.0 × 10^−4^, 3.0 × 10^−2^–5.0 × 10^−2^, 6.0 × 10^−3^–3.0 × 10^−2^, 3.0 × 10^−22^–6.0 × 10^−22^, 8.0 × 10^−6^–1.0 × 10^−5^, 8.0 × 10^−11^–1.0 × 10^−10^, 3.0 × 10^−3^–6.0 × 10^−2^, 8.0 × 10^−4^–6.0 × 10^−4^, 3.0 × 10^−4^–2.0 × 10^−4^, 2.0 × 10^−4^–1.0 × 10^−4^, 2.0 × 10^−2^–2.0 × 10^−1^, 2.0 × 10^−4^–4.0 × 10^−3^, 3.0 × 10^−30^–6.0 × 10^−29^, 2.0 × 10^−3^–1.5 × 10^−3^, 2.0 × 10^−9^–3.0 × 10^−9^, and 4 × 10^−3^–1.0 × 10^−2^, respectively. Similarly, the mean ± SD values for the aforementioned mine water variables were 1.0 × 10^−4^ ± 1.0 × 10^−6^, 1.0 × 10^−4^ ± 2.0 × 10^−7^, 4.0 × 10^−2^ ± 3.0 × 10^−2^, 1.0 × 10^−3^ ± 2.0 × 10^−4^, 5.0 × 10^−22^ ± 1.0 × 10^−22^, 9.0 × 10^−6^ ± 1.0 × 10^−6^, 9.0 × 10^−11^ ± 1.0 × 10^−11^, 4.0 × 10^−4^ ± 1.0 × 10^−2^, 1.0 × 10^−3^ ± 3.0 × 10^−4^, 4.0 × 10^−4^ ± 2.0 × 10^−4^, 4.0 × 10^−4^ ± 2.0 × 10^−4^, 5 × 10^−2^ ± 6.0 × 10^−3^, 3.0 × 10^−3^ ± 6.0 × 10^−2^, 5.0 × 10^−30^ ± 1.0 × 10^−30^, 4.0 × 10^−3^ ± 1.0 × 10^−4^, 2.0 × 10^−9^ ± 3.0 × 10^−10^, and 8.0 × 10^−4^ ± 2.0 × 10^−4^, respectively. The highest concentrations of dissolved ions were reported for Pb^4+^, Pb^2+^, Ni^2+^, Mn^2+^, Cd^2+^, Cu^+^, Cu^2+^, Fe^2+^, and Zn^2+^, respectively. The dissolved chemical ions in the groundwater of the present study were compared with the study reported by Rashid et al., 2021, which recorded a little lower concentration, attributed to the sufficient dilution from the Riverine water of River Swat [6]. However, comparing the groundwater and mine water profiles suggested that both waters are saturated from the same parent rock that was situated in the underlying bedrock settings. However, mine water showed higher concentrations than shallow water, mid-depth, and deep groundwater due to mining actions and human inputs.

### 3.5. Mineral Phases of Potentially Harmful Elements in Groundwater

Table 3 describes minerals phases for Ni, Mn, Cr, Cu, Cd, Pb, Co, Fe, and Zn in the groundwater system and mine water of the Adenzai flood plain region. The mineral composition of the groundwater system shows that the mean ± SD values for bunsenite, Ni(OH)_2_, trevorite, birnessite, bixbyite, hausmannite, manganite, manganosite, pyrolusite, todorokite, chromite, eskolaite, CuCr_2_O_4_, cuprite, delafossite, ferrite-Cu, tenorite, monteponite, crocoite, litharge, massicot, minium, plattnerite, spinel-Co, goethite, hematite, magnetite, wustite, ferrite-Zn, zincite, and ZnCr_2_O_4_ were −2.3 ± 0.308, −2.6 ± 0.308, 17.0 ± 0.803, −56.0 ± 2.038, −7.7 ± 0.509, −8.5 ± 0.763, −4.2 ± 0.254, −7.7 ± 0.255, −9.3 ± 0.255, −44 ± 4.52, 19.8 ± 0.595, 19.8 ± 0.595, 17 ± 0.365, 18.3 ± 0.616, 5.38 ± 0.943, 16.5 ± 0.674, 16.3 ± 0.957, 2.34 ± 0.463, −5.5 ± 0.363, −5.5 ± 0.55, −3.3 ± 0.471, −3.5 ± 0.491, −23.0 ± 1.426, −18 ± 0.465, −8.9 ± 1.071, 7.42 ± 2.512, 16.5 ± 0.67, 16.5 ± 1.006, −2.8 ± 0.317, 15.1 ± 0.696, −1.0 ± 0.166, and 26.9 ± 0.393, respectively. Likewise, the mean ± SD concentrations of the aforementioned minerals in the mine water were −2.09 ± 0.07, −2.37 ± 0.06, 18.5 ± 0.11, −51.4 ± 0.71, −6.59 ± 0.18, −6.91 ± 0.27, −3.62 ± 0.09, −7.13 ± 0.1, −8.77 ± 0.09, −40.5 ± 0.63, 21.0 ± 0.11, 17.5 ± 0.1, 19.8 ± 0.11, 7.06 ± 0.18, 7.06 ± 0.18, 17.9 ± 0.1, 18.4 ± 0.13, 3.19 ± 0.09, −4.69 ± 0.05, −4.29 ± 0.13, −2.3 ± 0.1, −2.48 ± 0.1, −20.4 ± 0.31, −16.9 ± 0.1, −6.75 ± 0.28, 8.41 ± 0.05, 17.8 ± 0.1, 9.16 ± 17.0, −2.26 ± 0.03, 16.8 ± 0.09, −0.52 ± 0.1, and 27.9 ± 0.17, respectively. The means ± SD of T. hydrogen, T. oxygen, and ionic strength in the groundwater system were 111.0 ± 0.004, 55.5 ± 0.044, and 0.005 ± 0.001, and the means ± SD of mine water were 111.0 ± 0.04, 55.5 ± 0.02, and 0.02 ± 0.01, respectively. The comparative study of groundwater and mine water revealed that mine water is more saturated due to dissolution and a higher interaction time with underground water. Thus, overall, the highest mean ± SD concentration of minerals was observed in the mine water.

### 3.6. Non-Carcinogenic and Carcinogenic Risk of PHEs

**Figure 5:**
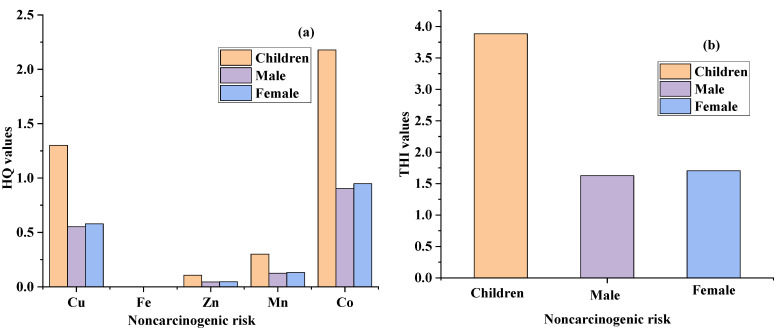

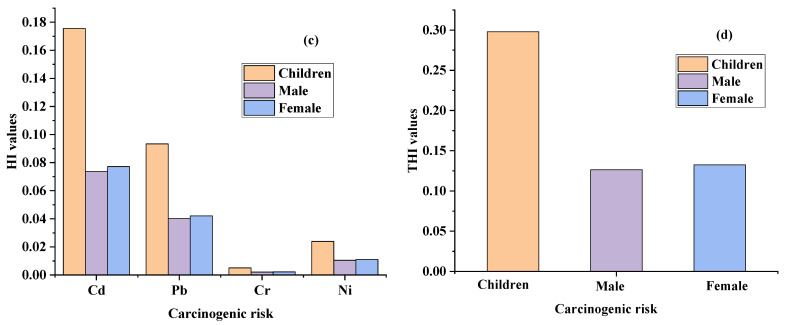
(**a**) Represents HQ values of Cu, Fe, Zn, Mn, and Co for children, male, and female exposed to noncarcinogenic risk, (**b**) THI values of noncarcinogenic risk, (**c**) HI values for Cd, Pb, Cr, and Ni causes carcinogenic risk, and (**d**) THI values of carcinogenic risk in children, male, and female in Adenzai flood plain region of Pakistan.

The non-cancer risk posed by PHEs, viz., Mn, Cu, Co, Fe, and Zn, in children, males, and females, is described in Figure 5 and Table 4. The ranges of concentrations of the non-cancer risk posed by PHEs, such as Mn, Cu, Co, Fe, and Zn, in children were 0.044–1.25, 0.03–6.76, 0.102–9.63, 2 × 10^−5^–0.002, and 0.041–0.3 mg/Kg·day, in males they were 0.018–0.52, 0.012–2.81, 0.042–4.0, 7 × 10^−6^–0.001, and 0.017–0.14 mg/Kg·day, and in females they were 0.019–0.55, 0.013–2.94, 0.044–4.19, 8 × 10^−6^–0.005, and 0.018–0.15 mg/Kg·day, respectively. In contrast, the ranges of concentrations of THI in children, males, and females were 0.217–17.9, 0.09–7.47, and 0.094–7.83 mg/Kg·day, respectively. The non-cancer risk suggested that children are more severely influenced by PHE ingestion than males and females in the study area. The results of the cancer risk posed by PHEs, viz., Ni, Cr, Cd, and Pb, in children and adults are outlined in Figure 5 and Table 4.

The ranges of concentrations posed by PHEs in children, due to Ni, Cr, Cd, and Pb, were 1.0 × 10^−7^–5.0 × 10^−2^, 6.0 × 10^−4^–2.0 × 10^−2^, 1.7 × 10^−2^–8.0 × 10^−1^, and 9.0 × 10^−3^–5.5 × 10^−1^ mg/Kg·day, in males they were 1.0 × 10^−3^–2.0 × 10^−2^, 2.0 × 10^−4^–1.0 × 10^−2^, 7.0 × 10^−3^–3.3 × 10^−1^, and 0.004.0 × 10^−3^–2.3 × 10^−1^ mg/Kg·day, and in females they were 1.0 × 10^−3^–2.0 × 10^−2^, 2.0 × 10^−4^–1.0 × 10^−2^, 7.0 × 10^−3^–3.5 × 10^−1^, and 4.0 × 10^−3^–2.4 × 10^−1^ mg/Kg·day, respectively. However, the ranges of concentrations of THI in children, males, and females were 2.7 × 10^−2^–14.2 × 10^−1^, 1.3 × 10^−2^–5.9 × 10^−1^, and 1.3 × 10^−2^–6.2 × 10^−1^ mg/Kg·day, respectively. The cancer risk suggested that children are heavily influenced by PHE intake from groundwater in the entire area. The increasing trend of cancer severity shows children > female > male. Moreover, the increasing order of PHEs recorded for non-carcinogenic and carcinogenic risks was observed as Co > Mn > Zn > Cu > Fe, and Cd > Pb > Cr > Ni, respectively. The groundwater health risk study was outlined, and Ni and Cd for children were above the safe limit (HRI < 1), suggesting a possible health risk in this region. The comparative study suggested that the resulting non-carcinogenic and carcinogenic health impacts for Ni, Mn, Cr, Cu, Cd, Pb, and Zn were higher than those reported by Aghlmand et al., 2021, Muhammad et al., 2010, and Shah et al., 2012 [37]. Thus, noncarcinogenic and carcinogenic health concerns were evident in the entire study area.

### 3.7. Pearson Correlations for the Interrelationship of Measured Ions and Trace Metals

The Pearson correlation matrix of PHEs and physicochemical variables in the groundwater samples were listed (see Table S1). The interrelationship of various water variables had significant positive and negative impacts, measuring the degree of closeness and establishing a linear relationship between the dependent and independent groundwater variables. The correlation coefficient (r) mainly indicated the merging of the sampling points close in a straight line, such as the coefficient of determination (R^2^) values of Cr, Cd, and Pb, respectively. The inter-elemental analysis favored the results of the principal component analysis, multilinear regression (PCA-MLR).

The most significant correlation coefficient values were observed for the following correlating pairs: pH and EC (r = 0.70), pH and TDS (r = 0.72), pH and Na (r = 0.70), pH and HCO_3_ (r = 0.72), pH and SO_4_ (r = 0.73), EC and TDS (r = 0.98), Na and EC (r = 0.83), EC and HCO_3_ (r = 0.81), EC and SO_4_ (r = 0.83), EC and Mn (r = 0.75), EC and Cr (r = 0.71), and EC and Cd (r = 0.79), respectively, whereas, the following pair values were Na and TDS (r = 0.84), TDS and HCO_3_ (r = 0.81), TDS and SO_4_ (r = 0.84), TDS and Cd (r = 0.80), TDS and Pb (r = 0.77), TDS and Co (r = 0.76), and TDS and Zn (r = 0.72), respectively. However, the following pair values were Ca and Mg (r = 0.72), Ca and Na (r = −0.71), Ca and HCO_3_ (r = −0.70), Mg and Na (r = −0.76), and Mg and HCO_3_ (r = −0.72), (r = 0.70), respectively. The following pair values were Na and HCO_3_ (r = 0.91), Na and SO_4_ (r = 0.90), Na and Mn (r = 0.72), Na and Cd (r = 0.77), Na and Pb (r = 0.73), Na and Fe (r = 0.74), Na and Zn (r = 0.70), HCO_3_ and SO_4_ (r = 0.84), HCO_3_ and Cd (r = 0.72), HCO_3_ and Pb (r = 0.74), SO_4_ and Mn (r = 0.82), SO_4_ and Cu (r = 0.70), SO_4_ and Cd (r = 0.83), SO_4_ and Co (r = 0.74), and SO_4_ and Zn (r = 0.76), respectively, whereas the following pair values were Mn and Cr (r = 0.80), Mn and Cd (r = 0.82), Mn and Pb (r = 0.84), Mn and Co (r = 0.80), Mn and Fe (r = 0.85), and Mn and Zn (r = 0.72), respectively. The following pair values were Cr and Cd (r = 0.78), Cr and Pb (r = 0.83), Cu and Cd (r = 0.70), Cd and Pb (r = 0.84), Cd and Co (r = 0.78), Cd and Fe (r = 0.82), Cd and Zn (r = 0.74), Pb and Co (r = 0.82), Pb and Fe (r = 0.80), Co and Fe (r = 0.74), and Fe and Zn (r = 0.73), respectively.

The PHE pairs of Cr–Ni, Cr–Mn, Cr–Cd, Cr–Pb, and Cr–Fe correlated significantly at the *p* < 0.05 level. The coefficient of determination (R^2^) values of Cr versus Ni, Mn, Cu, Cd, Pb, Co, Fe, and Zn were 0.07, 0.04, 0.04, 0.02. 0.15, 0.07, 0.25, and 1.14 × 10^−4^, respectively (see Figure S1). Similarly, the coefficient of determination (R^2^) values of Cd versus Ni, Mn, Cr, Cu, Pb, Co, Fe, and Zn were 0.02, 0.04, 0.01, 0.13, 0.002, 0.12, and 1.79 × 10^−4^, respectively (see Figure S2). Similarly, the coefficient of determination (R^2^) values of Pb versus Ni, Mn, Cr, Cu, Pb, Co, Fe, and Zn were 0.01, 0.009, 0.15, 0.09, 0.13. 0.006, 0.06, and 0.009, respectively (see Figure S3). Similarly, the coefficient of determination (R^2^) values of northing versus Ni, Mn, Cr, Cu, Cd, Pb, Co, and Zn were 0.03, 0.12, 2.963 × 10^−4^, 0.05, 0.004, 0.004, 0.07, and 0.001, respectively (see Figure S4).

It is worth noting that higher concentrations of Cr, Pb, Cd, Fe, and Co were observed in most areas of the study area. This is not surprising because all of these contaminants are directly influenced by mining activities, coal combustion, and agriculture practices. Meanwhile, the acidic-to-neutral environment mostly favors the dissolution of PHEs. Outside of the mining areas, the groundwater is influenced by natural and anthropogenic activities.

### 3.8. Groundwater Pollution Indexing in Complex Water Aquifer

Table S2 describes the pollution indexes for PHEs in the groundwater system of Adenzai, northern Pakistan. The pollution indexes of Ni, Mn, Cr, Cu, Cd, Pb, Co, Fe, and Zn were 1.14, 1.34, 1.79, 2.80, 1.75, 3.36, 2.11, 2.09, and 0.97. The pollution index indicated that these contaminants showed moderate pollution in the entire region. The highest contamination values were recorded for Cr, Cu, Cd, Pb, Co, and Fe up to 1.79, 2.80, 1.75, 3.36, 2.11, and 2.09 in the study area. The groundwater contamination resulted from the weathering and dissolution of mafic and ultramafic rock, mining action, steel industrial effluents, agriculture practices, and transportation sectors. The transportation sector extensively uses leaded gasoline and petrol for vehicular transportation. However, the increasing order contamination factors was described as Pb > Cu > Co > Fe > Cr > Cd > Mn > Ni > Zn, respectively. The overall groundwater quality revealed moderate contamination in the entire region.

### 3.9. Spatial Distribution of Groundwater Variables and PHEs Using Q-Q Plotting

Groundwater physicochemical variables and PHEs were used to construct Q-Q plotting to determine normal distribution. This is a graphical way of plotting data from the first data set’s quantiles against the second data set. The first data set is labeled “anticipated expected normal value”, plotted against the second data set denoted by “observed values”. These numbers align up and down at the 45° reference line plotting data in SPSS software. The quantile values were arranged in a straight diagonal line. If both data sets came from the same distribution source, this reference line would be linear (normal distribution).

The findings of groundwater Q-Q normal standard plots are described in (see Figure 5). The groundwater data set shows the normal distribution for some observations, but few data deviated significantly by adopting a curved pattern away from a straight reference line. The deviated observations are evident by occupying the extreme points known as outliers. Q-Q plotting determined a normal distribution and the outliers in the groundwater samples. The groundwater was assembled within three quartiles, viz., Q1, Q2, and Q3. First quartile Q1 was the lower quartile, Q2 was the median, and Q3 was the upper quartile. The numerical values of groundwater data occurred below the median occupied in the first quartile, and the higher-concentration values were reported in the Q3 upper quartile. However, the normal values of groundwater samples were assembled in the second quartile Q2. The lower quartile arranged groundwater samples in increasing sequence within 25%. The upper quartile Q3 accumulated groundwater data points and accounted for 75%. Thus, normal distribution in groundwater data was reported in the Q2-median quartile that occurred between the Q1 and Q3 quartiles in the study area.

The quantile of one data set (anticipated normal values) vs. the second data set (observed values) recorded a straight reference line at 45 degrees in the normal Q-Q box plot of the physicochemical variables, viz., pH, EC, temperature, depth, Ca, Mg, K, Na, HCO_3_, Cl, SO_4_, and the PHEs, viz., Ni, Mn, Cr, Cu, Cd, Pb, Co, Fe, and Zn, in the groundwater of the Adenzai flood plain region, Pakistan. The correlation R^2^ values of the physicochemical variables, viz., pH, EC, temperature, depth, Ca, Mg, K, Na, HCO_3_, Cl, and SO_4_, were 0.990, 0.888, 0.956, 0.843, 0.852, 0.846, 0.988, 0.943, 0.876, 0.713, 0.983, and 0.838, and the values of the PHEs, viz., Ni, Mn, Cr, Cu, Cd, Pb, Co, Fe, and Zn were 0.958, 0.734, 0.796, 0.708, 0.734, 0.759, 0.743, 0.842, and 0.772, respectively (see Figure 5). In the groundwater data set, the maximum quantile and straightness of the reference line were found for pH, Mg, Cl, Ni, temperature, and K, and their correlation coefficient R^2^ values were 0.990, 0.988, 0.983, 0.958, 0.956, and 0.943, respectively. The increasing order of Q-Q normal distribution was recorded in the following sequence: pH > Mg > Cl > Ni > temperature > K > EC > Na > TDS > Ca > depth > SO_4_ > HCO_3_ > Fe > Cr > Zn > Pb > Co > Cd > Cu, respectively. The findings of the Q-Q normal distribution were compared and found consistent with the findings of Rashid et al., 2021 [6].

### 3.10. Nemerow’s Pollution Indexing (NPI)

Table 5 represents the ranges of values of NPI tested for groundwater parameters, viz., pH, EC, TDS, Ca, Mg, K, Na, HCO_3_, Cl, SO_4_, Ni, Mn, Cr, Cu, Cd, Pb, Co, Fe, and Zn. The ranges of values of NPI in the shallow groundwater were 0.85–0.98, 0.85–0.98, 0.21–0.8, 0.27–1.0, 0.3–0.66, 0.38–1.58, 0.28–1.75, 0.7–2.83, 0.32–0.6, 0.23–0.48, 0.02–0.18, 0.11–1.0, 0.32–0.6, 0.02–0.95, 0.2–6.2, 1.0–20.0, 0.14–6.0, 0.77–4.47, and 0.04–0.22, those for mid–depth groundwater were 0.824–0.95, 0.833–2.58, 0.21–0.64, 0.34–0.85, 0.36–0.74, 0.375–0.9, 0.225–0.85, 0.6.0–1.12, 0.22–0.58, 0.179–0.47, 0.014–0.18, 0.16–1.0, 0.22–0.58, 0.015–0.23, 0.222–4.6, 1.0–16.1, 0.75–5.75, 0.8–5.2, and 0.04–0.12, those for deep groundwater were 0.847–0.95, 1.174–2.8, 0.3–0.68, 0.28–1.2, 0.36–0.9, 0.075–0.9, 0.11–0.75, 0.633–1.1, 0.32–0.54, 0.158–0.3, 0.011–0.13, 0.16–0.7, 0.32–0.54, 0.005–0.12, 0.2–1.2, 1.0–4.0, 0.75–5.25, 0.367–1.03, and 0.04–0.12, and those for mine water were 0.89–0.96, 4.13–4.63, 1.05–1.28, 0.2–0.29, 0.2–0.42, 0.15–1.0, 1.68–2.05, 2.03–2.27, 0.32–0.48, 0.65–0.7, 0.11–0.17, 1.3–3.16, 0.32–0.48, 0.6–1.13, 7.0–9.6, 25–58, 6.0–13, 7–9.5.0, and 0.15–0.3, respectively. However, the mean ± SD values of NPI in the shallow groundwater for the aforementioned parameters for shallow groundwater were 0.9 ± 0.03, 1.85 ± 0.66, 0.46 ± 0.16, 0.41 ± 0.15, 0.51 ± 0.09, 0.75 ± 0.25, 0.81 ± 0.32, 1.02 ± 0.41, 0.46 ± 0.08, 0.33 ± 0.07, 0.08 ± 0.05, 0.62 ± 0.26, 0.46 ± 0.08, 0.24 ± 0.23, 1.49 ± 1.51, 6.88 ± 6.04, 1.78 ± 1.48, 2.88 ± 1.25, and 0.08 ± 0.04, those for mid-depth water were 0.87 ± 0.04, 1.69 ± 0.53, 0.41 ± 0.13, 0.55 ± 0.16, 0.59 ± 0.12, 0.69 ± 0.21, 0.47 ± 0.21, 0.86 ± 0.18, 0.38 ± 0.08, 0.3 ± 0.07, 0.07 ± 0.05, 0.49 ± 0.31, 0.38 ± 0.08, 0.07 ± 0.06, 1.4 ± 1.32, 7.09 ± 5.61, 2.29 ± 1.39, 2.35 ± 1.69, and 0.07 ± 0.02, those for deep groundwater were 0.89 ± 0.03, 1.82 ± 0.54, 0.45 ± 0.13, 0.67 ± 0.31, 0.57 ± 0.17, 0.51 ± 0.29, 0.43 ± 0.22, 0.88 ± 0.15, 0.42 ± 0.07, 0.26 ± 0.05, 0.06 ± 0.04, 0.45 ± 0.19, 0.42 ± 0.07, 0.06 ± 0.04, 0.72 ± 0.29, 1.75 ± 1.06, 1.88 ± 1.36, 0.78 ± 0.22, and 0.08 ± 0.02, and those for mine water were 0.93 ± 0.02, 4.5 ± 0.17, 1.13 ± 0.1, 0.25 ± 0.03, 0.3 ± 0.07, 0.48 ± 0.32, 1.84 ± 0.13, 2.17 ± 0.09, 0.38 ± 0.06, 0.67 ± 0.02, 0.14 ± 0.03, 2.29 ± 0.65, 0.38 ± 0.06, 0.75 ± 0.2, 8.2 ± 1.01, 42.6 ± 12.3, 10.0 ± 2.45, 8.4 ± 0.97, and 0.23 ± 0.05, respectively.

The results obtained after the calculation of groundwater sources for NPI are considered alarming if the NPI is greater than 1 and/or NPI > 1. The aforementioned results of NPI declared that the groundwater sources were highly contaminated with EC, Ca, K, Na, HCO_3_, Mn, Cd, Pb, Co, and Fe, and their percentage contributions were 90%, 12%, 10%, 38%, 70%, 22%, 52%, 100%, 94%, and 86%; these samples had exceeded the allowable limit of 1.0. Moreover, in shallow groundwater, the exceeded variables revealed 91.6%, 4.1%, 8.3%, 29.2%, 66.6%, 12.5%, 41.6%, 100%, 66.6%, 91.6%, in mid-depth water they were 87.5%, 7.1%, 14.2%, 14.2%, 42.8, 7.1% 42.8%, 100%, 92.8%, 78.5%, and in deep groundwater they were 100%, 33.3%, 8.3%, 25%, 50%, 7.1%, 25%, 100%, 91.6%, and 25%, respectively. However, the exceeded parameters in mine water included EC, Na, HCO_3_, Mn, Cd, Pb, Co, and Fe, and their percent contribution was 100% for each parameter reported above. The groundwater results of the present study for NPI were compared with the findings of [57]. The results of this study revealed that the findings were consistent and result-oriented.

### 3.11. Cluster Analysis

**Figure 6:**
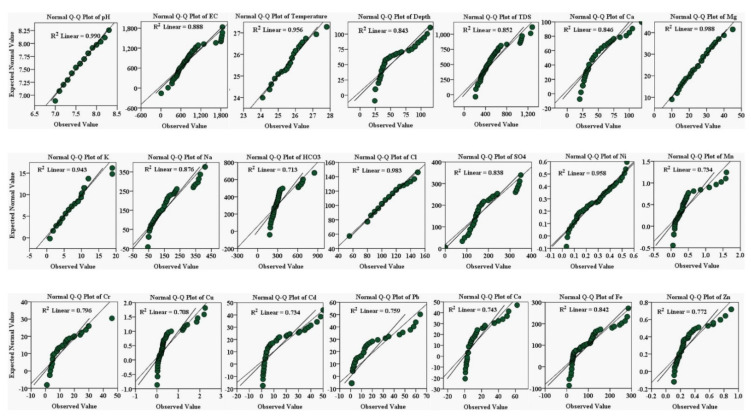
Describe the normal Q-Q box plot of physicochemical variables, viz., pH, EC, temperature, depth, Ca, Mg, K, Na, HCO_3_, Cl, SO_4_, and the PHEs, viz., Ni, Mn, Cr, Cu, Cd, Pb, Co, Fe, and Zn, in the groundwater of the Adenzai flood plain region, Pakistan.

**Figure 7:**
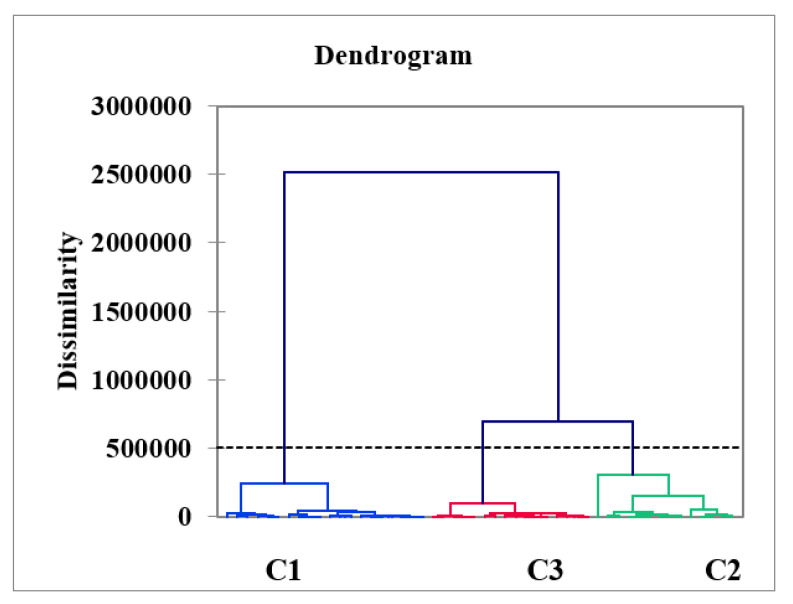
Potential clusters described after Ward’s classification that grouped PHEs into three classes, C1, C2, and C3, in the Adenzai area.

Cluster analysis is a valuable technique to represent the groundwater data in clusters/groups. It helped in understanding the groundwater data and in displaying patterns of the most similar objects. According to this technique, the most likely observations fell within the same class/category [69]. The most similar observations were arranged in three different classes, which were classes C1, C2, and C3 (see Figure 6). Overall, the results of the clustering analysis (CA) and PCA-MLR showed three major processes. Through these processes, different classes/groups were constructed to form a dendrogram [70]. The targeted groundwater observations were tested through Ward’s method, and the squared Euclidean distance was measured for the similarity index. Class C1 represents 17 groundwater samples from Badwan, Chatpat, Ramial, and Osaky (5, 4, 3, and 5). The second class was composed of twenty-nine (*n* = 29) groundwater samples from Badwan, Chatpat, Ramial, Osaky, Warsak, and Rambora areas (3, 2, 5, 3, 8, and 8, respectively). The third class, C3, represented two groundwater samples from Chatpat. Cluster C1 was considered less polluted, and the %age contribution was 60.4%. Class C2 showed moderate pollution, and the %age contribution was 36%. Class C3 revealed severe pollution, and the %age contribution was 4.2%. However, variability was 41.6% within the class, and between the class it was 58.4% (see Figure 6). The range and mean centroid distances between the three classes, C1, C2 and C3, were (0.17–0.69 and 0.39), (0.28–0.81 and 0.43), and (0.25–0.25 and 0.25), respectively. However, the groundwater samples were grouped into clusters, and C1, C2, and C3 were classified as low, medium, and high polluted areas corresponding to the PHE concentrations. The overall groundwater observation was expressed via the clustering plot (see Figure 7 and Figure S5).

### 3.12. Pollution Source Identification

**Figure 8:**
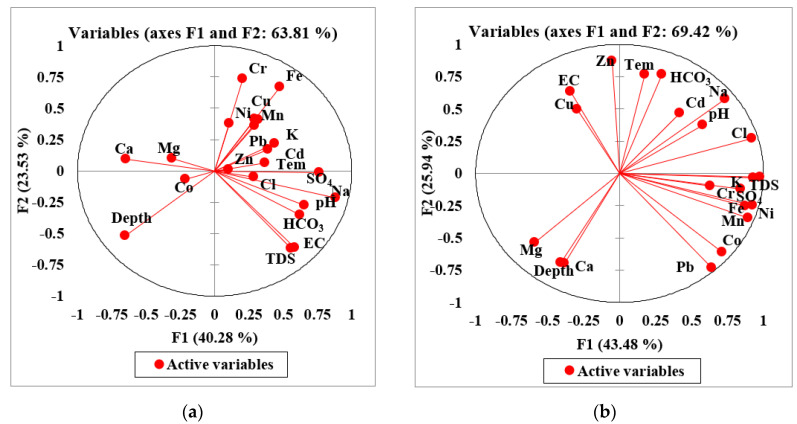
(**a**) PCA results of the first two factors FI and F2 in the groundwater and (**b**) mine water of the study area.

The principal component analysis multi-linear regression was performed on the normalized groundwater data. This approach determined the pollution sources in terms of %age contribution. Once the pollution source was identified, it was easy to minimize its impact on groundwater. These studies mainly focused on loading factors to define hydrological processes for particular areas [71]. It calculated three significant loading factors, F1, F2, and F3, respectively. The PCA-MLR results of fifty groundwater samples were described for nineteen groundwater variables. The overall loading factors and the correlation between the first and second loading factors are described in Figure 8. However, the calculated loading factors, F1, F2, and F3, cumulative %age (80.223), and individual factor variance based on percentage were 40.28, 23.53, and 16.41 and are listed in Table 6 and Figure 8a. Mine water representing F1, F2, and F3 loading factors, their cumulative %age (85.452), and individual factor variance were 43.482, 25.936, and 16.034 (see Table 6, and Figure 8b).

The first loading factor, F1, showed a 40.285 % variability with the eigenvalues of 4.95 (see Figure 8). The significant variables of F1 were pH, EC, depth, TDS, Ca, Mg, Na, HCO_3_, SO_4_, Mn, Cd, and Pb, and their correlating coefficient values were 0.658, 0.561, −0.652, 0.585, −0.648, −0.512, 0.883, 0.623, 0.762, 0.523, 0.541, and 0.520, respectively (see Table 6). The component F1 showed ionic strength, major ion constituents, bedrock composition, and groundwater rock interaction, suggesting that groundwater contamination takes its inception from the weathering of granite rocks, ion exchange, and the dissolution of minerals in the entire region. Mostly, the groundwater variables of factor F1 revealed the genesis of the geogenic source. However, pH, EC, and TDS resulted from mineral dissolution, the leakage of aquifer compositions, and saline bases [72,73,74], whereas Ca^2+^, Na^2+^, HCO_3_^–^, and SO_4_^2−^ originated from geogenic sources, i.e., ion exchange processes, rock weathering, water-rock interaction, and dissolution of albite muscovite, and dolomite minerals. The potential sources contributing to Ca^2+^, Na^2+^, and SO_4_^2−^ were attributed to be calcite (CaCO₃), plagioclase (NaAlSi_3_O_8_–CaAl_2_Si_2_O_8_), and sulfate bearing minerals such as gypsum polyhalite (K_2_Ca_2_ Mg (SO_4_)_6_, (CaSO_4_·2H_2_O). However, the sources of HCO_3_^–^ in groundwater take their genesis from water transportation, and from interactions with calcium or magnesium carbonate rocks containing dolomite and limestone-forming bicarbonates. The potential sources of Mn were soil weathering, organic matter, gasoline, compost, silage pile, and bedrock composition collectively released into the water aquifer, while Cd in the groundwater system were triggered from shale, fossil fuels, and coal during volcanic action. However, Pb takes its genesis from the lead pipe, plumbing materials, corrosion, and pipe fixture. The loading factor F1 determined that geogenic inputs accounted for 75% of contribution in groundwater (see Figure 8).

**Figure 9:**
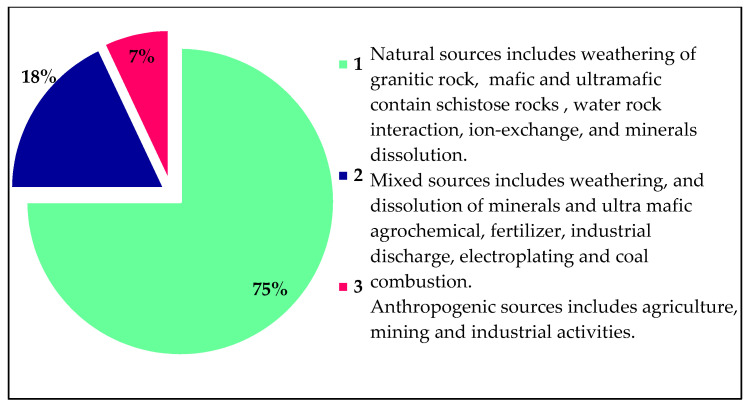
The percentage of contributions of pollution sources of groundwater in Adenzai, the flood plain region, Pakistan.

The second loading factor showed a 23.526 % variance with the eigenvalues of 2.985 (see Figure 8). This factor contributed favorable loading for Ni, Cr, Cu, and Fe with correlation coefficient (r) values of 0.547, 0.734, 0.515, and 0.671. However, the negative loading observed for EC, depth, and TDS showed correlation coefficient (r) values that were −0.623, −0.521, and −0.613, respectively (Table 6 and Figure 8). The mineral phases suggested that Ni contamination in the water aquifer takes its origin from underlying bedrock materials, viz., bunsenite NiO, Ni (OH)_2_, and trevorite minerals. Cr contamination in the groundwater system takes its genesis from chromite FeCr_2_O_4_ and eskolaite Cr_2_O_3_ minerals, while Cu, Cr, and Fe get into the water through water–rock interaction with CuCr_2_O_4_, delafossite CuFeO_2_, and Cu-Ferrite CuFe_2_O_4_. However, Fe get into groundwater through interaction and the dissolution of goethite FeO(OH), hematite Fe_2_O_3_, magnetite Fe_3_O_4_, and wustite FeO minerals. Thus, EC, depth, and TDS negated values reflected that these water variables have an inverse correlation with water variables and loading factors. However, a negative loading in PCA essentially signified that those specific properties of water are missing a latent variable associated with the main PCA component. Additionally, groundwater variables were attributed to industrial discharge, electroplating, agrochemical fertilizer, and coal combustion [75]. The second loading factor determined mixed sources contributing natural and anthropogenic inputs in groundwater by accounting for 18% contribution (see Figure 9).

The third loading factor, F3 contributed 16.412% variability with the eigenvalues of 1.826 (see Table 6). The significant positive variables of F3 were Cl and Co, with correlating coefficient (r) values of 0.714 and 0.586, respectively. At the same time, Ni showed a negative correlation, and its correlating coefficient (r) value was −0.527 (see Table 6). The Cl got into the groundwater system from mineral deposits, seawater spray, industrial waste, and agriculture runoff. However, Co took its origin from mafic, ultramafic, and metamorphic rocks containing cobaltite and sulfosalt minerals. The third factor showed that anthropogenic pollution can play a vital role in the contamination of groundwater systems. The significant loading represents anthropogenic inclusion from industrial and agricultural activities, domestic wastes, poultry effluents, and Zn fertilizer [30]. The Ni gets into the water system through the weathering and erosion of mafic, ultramafic rock, water-rock interaction, and mineral dissolution [75,76]. The loading factor F3 recorded anthropogenic sources accounting for 7% of contribution (see Figure 8).

The PCA results of mine water were also described in three factors: F1, F2, and F3, with a total variability of 85.452 with the eigenvalues of 11.434 (see Table 6). The first loading factor, F1, revealed a 43.482% variability with the eigenvalues of 6.137 (see Figure 8b). The positive significant loading noticed for pH, EC, TDS, K, Na, Cl, SO_4_, Ni, Mn, Cr, Pb, Co, and Fe, had corresponding correlating coefficient values of 0.579, 0.643, 0.594, 0.932, 0.733, 0.921, 0.842, 0.923, 0.892, 0.633, 0.642, 0.714, and 0.873, and the negative loading for Ca^2+^ and Mg^2+^ had correlating coefficient values of −0.540 and −0.588, respectively (see Table 6). The groundwater contamination was evident from the saline water intrusion, leaking of aquifer composition, ion exchange, weathering and erosion of mafic and ultramafic rock, geochemical modification of sulfide minerals, water–rock interaction, and mineral dissolution [75,76]. Moreover, mineral phases of the current study proposed that Ni contamination could take its genesis from underground geological settings containing bunsenite NiO, Ni (OH)_2_, trevorite minerals, and Cr resulting from chromite FeCr_2_O_4_ and eskolaite Cr_2_O_3_. However, Fe may get into groundwater through the interaction and dissolution of goethite FeO(OH), hematite Fe_2_O_3_, magnetite Fe_3_O_4_, wustite FeO, Delafossite CuFeO_2_, and Cu-Ferrite CuFe_2_O_4_ minerals, whereas Ca^2+^ and Mg^2+^ could result from calcite and dolomite minerals.

The second loading factor, F2, of mine water showed 25.936% variability with the eigenvalues of 3.285 (see Figure 8b). This factor revealed positive loading for temp, Na, HCO_3_, Cu, Cd, and Zn with correlation coefficient (r) values of 0.764, 0.579, 0.765, 0.498, 0.464, and 0.871, and negative loading for depth, Ca, Mg, and Co with values of −0.694, −0.696, −0.735, and −0.609, respectively. The possible contamination source of temperature could be solar radiation, sunlight, and industrial pollution releasing greenhouse gases, CO_2_, and other gases, whereas Na^+^ originates from granitic rock, and HCO_3_ takes genesis from sedimentary rock contains minerals of calcite and dolomite. However, Cu in mine water is attributed to a sedimentary rock containing azurite, bornite, chalcocite, cuprite, and malachite minerals. In comparison, Cd gets into the water through water–rock interaction with cadmoselite, greenockite, and otavite minerals. In contrast, Zn is attributed to smithsonite, sphalerite, hemimorphite, zincite, hydrozincite, and willemite. However, the mineral phases suggested the following minerals for Cu to be cuprite, delafossite, Cu-ferrite, tenorite, Cd (monteponite), Zn from ferrite-Zn, and zincite (present study). Moreover, depth, Ca, Mg, and Co showed negative values, reflecting that these water variables have an inverse correlation with water variables and loading factors. However, a negative loading in PCA essentially signifies that those specific properties of water are missing a latent variable associated with the main PCA component. However, Ca and Mg could take their genesis from calcite and dolomite minerals, and Co could be attributed from serpentinite, dunite, and basalt mineral deposits. Cobalt is also influenced by cobaltite, a sulfosalt mineral containing cobalt, sulfur, and arsenic found in metamorphic rocks [77].

The third loading factor, F3 of mine water, determined a 16.034% variability with the eigenvalues of 2.012 (see Table 6). The favorable loading attributed for pH, temp, Cr, and Cd had correlation coefficient (r) values recorded as 700, 0.602, 0.718, and 0.751, and the negative loading for HCO_3_ was −0.562, respectively. The possible contamination source of F3 in mine water could be a geochemical modification of sulfide minerals. Additionally, groundwater variables are attributed to industrial discharge, electroplating, agrochemical fertilizer, and coal combustion [75], whereas Cd could be influenced by the weathering of schistose rocks, but it mainly occurs in sulphide minerals.

### 3.13. Implication for the Sustainable Management of Groundwater Resources

Groundwater contains several essential elements such as K^+^, Ca^++^, Mg^++^, S^2−^, Cl^−^, Ni, Cu, Mn, Fe, and Zn required for human health and body growth [66,78]. Most of these essential elements are required in a specific amount included in our daily food nutrition [79]. However, the excessive intake of the heavy elements interchangeably used with PHEs can cause health implications due to their persistent, genotoxic, and bioavailable nature. Moreover, PHEs can easily enter the food chain, interacting with the marine and terrestrial life that probably migrate and transport water contaminants to the ecosystem [80]. Thus, pollutants can easily transfer from one biological system into another, causing potential health risks to humans and the ecosystem [81]. However, the excessive ingestion of PHE implication for the sustainable management of groundwater resources is considered harmful to human health. The lower to moderate PHE ingestion poses the following diseases in human beings: hearing loss, lower intelligence, flu, growth retardation, and gastrointestinal problems [82], while excessive exposure to exceeded PHEs recorded irritability, sleep disturbances, vomiting, constipation, cramps, loss of appetite, exhaustion, convulsions, neurological impairment, coma, organ failure, and death [30,66]. Moreover, the carcinogenic and noncarcinogenic health implications recorded in the current study showed that children are more susceptible to PHEs than adults. Thus, PI and NPI were calculated to test the contamination level of PHEs, which indicated moderate to severe pollution in the entire region. This problem is extremely aggravated in the countries where alternate water sources are negligible and could increase the importance of groundwater for domestic, viz., cooking and drinking, purposes. Therefore, it is extremely important to monitor and safeguard the existing groundwater resources by preventing the further deterioration of water resources.

Our research strongly recommends that groundwater management and government authorities should take action to establish a monitoring network for groundwater systems. This can aid in the real-time monitoring of groundwater quality and availability and draw up plans to avoid groundwater deterioration. Local governments should push for more stringent steps to establish safe drinking water wells. Furthermore, appropriate initiatives should be implemented to raise public knowledge about the importance of using groundwater resources in a sustainable and safe manner.

## 4. Conclusions

The groundwater of Adenzai, the floodplain region is Pakistan, exceeded the WHO guideline values of EC, Ca^2+^, K^+^, Na^+^, HCO_3_^−^, Cr, Cd, Pb, Co, and Fe up to 90%, 18%, 16%, 28%, 16%, 60%, 44%, 78%, 76%, and 76%, respectively. Nemerow’s pollution index showed that EC, Ca, K, Na, HCO_3_, Mn, Cd, Pb, Co, and Fe had worse water quality, and their percentage exceedances were 90%, 12%, 10%, 24%, 56%, 10%, 38%, 100%, 80%, and 72%. Groundwater PHE contamination was more evident in the shallow aquifer, as compared to the mid-depth and deep aquifers, due to the weathering of granitic and gneissic rock and known existing mines. The mineral prospects of groundwater become precipitated due to supersaturation and dissolution, which is a result of undersaturation. PCAMLR and CA recorded that 75% of groundwater contamination takes it’s origin from geogenic sources, while 18% was from mixed and 7% was from anthropogenic sources. However, CA classified groundwater data into less, moderate, and severe polluted clusters. The HHRA-model suggested potential non-carcinogenic and carcinogenic risks in children and adults. The THI for non-carcinogenic and carcinogenic risks indicated that women and their infants are highly exposed to PHEs hazards. The higher THI values of the HHRA-model suggested that the groundwater sources were unfit for drinking and domestic needs. However, it is highly recommended that the government and non-government organizations play their role in minimizing the PHE concentration in the groundwater sources in the entire region and that they provide alternate sources of water for drinking and domestic purposes. Moreover, the use of agrochemical fertilizers and the consumption of mine water should also be reduced by raising awareness programs regarding the sustainable use and management of groundwater resources. Thus, all groundwater sources need immediate remedial measures to avoid public health concerns.

## Figures and Tables

**Table 1:** Statistical results of PHEs and physicochemical variables in the groundwater (*n* = 50) that were collected from different depths and mining areas of the Adenzai flood plain area, Pakistan.

Statistic	Shallow Water (*n* = 24)		Mid–Depth Water (*n* = 14)		Deep Water (*n* = 12)		Mine Water (*n* = 7)		WHO Limit
	Range	Mean ± SD	Range	Mean ± SD	Range	Mean ± SD	Range	Mean ± SD	
pH	7.2–8.3	7.6 ± 0.3	7.0–8.1	7.4 ± 0.3	7.2–8.1	7.5 ± 0.2	7.6–8.2	7.9 ± 0.2	6.5–9.2
EC µS/cm	212–1288	738.9 ± 263.0	333–1030	674.2 ± 212.1	469–1121	729.2 ± 217.7	1650–1850	1801.8 ± 69.3	400
Temp °C	24.5–26.6	25.7 ± 0.6	24.6–27.2	25.6 ± 0.6	24.5–26.2	25.5 ± 0.5	24.1–27.8	26.0 ± 1.3	-
Depth m	25.0–40.0	35.5 ± 4.3	41.0–80.0	56.2 ± 13.0	85.0–115.0	97.5 ± 9.4	24.0–35.0	28.7 ± 4.2	-
TDS mg/L	210–800	462.3 ± 156.8	210–635	412.9 ± 126.9	300–680	450.0 ± 127.8	1050–1280	1125.7 ± 96.4	1000
Ca mg/L	27–100	40.8 ± 15.4	34.0–85.0	54.8 ± 15.9	28.0–120.0	66.8 ± 31.0	20.0–29.0	24.7 ± 3.0	100
Mg mg/L	15.0–33.0	25.4 ± 4.3	18.0–37.0	29.5 ± 5.8	18.0–45.0	28.6 ± 8.4	10.0–21.0	15.1 ± 3.4	50
K mg/L	4.5–18.9	9.0 ± 2.9	4.5–10.8	8.3 ± 2.5	0.9–10.8	6.1 ± 3.4	1.8–12.0	5.7 ± 3.9	12
Na mg/L	55–350	162.3 ± 63.0	45.0–170.0	94.3 ± 42.4	22.0–150.0	86.3 ± 44.8	335–410	367.1 ± 25.1	200
HCO_3_ mg/L	210–850	307.3 ± 123.8	180–335	259.3 ± 52.9	190–330	263.3 ± 45.2	610–680	651.4 ± 27.9	500
Cl mg/L	80–150	114.2 ± 20.2	55.0–145.0	96.1 ± 20.9	80.0–135.0	103.8 ± 16.3	80–120	94.3 ± 15.4	250
SO_4_ mg/L	115–241	165.2 ± 34.4	89.3–236.3	151.9 ± 36.3	78.8–152.3	128.3 ± 23.1	325–350	337.0 ± 8.8	500
Ni mg/L	0.05–0.53	0.25 ± 0.14	0.04–0.54	0.22 ± 0.14	0.03–0.40	0.18 ± 0.13	0.32–0.52	0.41 ± 0.08	3.0
Mn mg/L	0.06–0.50	0.31 ± 0.13	0.08–0.50	0.24 ± 0.16	0.08–0.35	0.23 ± 0.10	0.65–1.58	1.15 ± 0.33	0.5
Cr mg/L	0.03–0.15	0.09 ± 0.04	0.03–0.17	0.07 ± 0.01	0.01–0.08	0.04 ± 0.02	0.19–0.30	0.24 ± 0.04	0.05
Cu mg/L	0.03–1.90	0.47 ± 0.45	0.03–0.45	0.15 ± 0.12	0.01–0.24	0.11 ± 0.08	1.20–2.25	1.50 ± 0.40	2.0
Cd mg/L	0.01–0.31	0.07 ± 0.08	0.01–0.23	0.07 ± 0.02	0.01–0.06	0.04 ± 0.01	0.35–0.48	0.41 ± 0.05	0.05
Pb mg/L	0.01–0.20	0.07 ± 0.06	0.01–0.16	0.07 ± 0.02	0.01–0.04	0.02 ± 0.01	0.25–0.58	0.43 ± 0.12	0.01
Co mg/L	0.01–0.24	0.07 ± 0.06	0.03–0.23	0.09 ± 0.03	0.03–0.21	0.08 ± 0.05	0.24–0.52	0.40 ± 0.10	0.04
Fe mg/L	0.23–1.34	0.86 ± 0.38	0.24–1.56	0.70 ± 0.51	0.11–0.31	0.23 ± 0.07	2.10–2.85	2.52 ± 0.29	0.3
Zn mg/L	0.11–0.65	0.23 ± 0.13	0.12–0.37	0.22 ± 0.07	0.12–0.35	0.25 ± 0.06	0.45–0.90	0.70 ± 0.15	3.0

**Table 2:** Chemical speciation of PHEs in groundwater and mine water in the Adenzai flood plain area, Pakistan.

Statistics	Groundwater (*n* = 50)		Mines Water (*n* = 7)	
	Range	Mean ± SD	Range	Mean ± SD
H^+^	1.0 × 10^−4^–1.15 ×10^−4^	1.0 × 10^−4^ ± 1.0 × 10^−6^	1.0 × 10^−4^–1.0 × 10^−4^	1.0 × 10^−4^ ± 1.0 × 10^−6^
HO^−^	1.0 × 10^−4^–1.05 × 10^−4^	1.0 × 10^−4^ ± 9.0 × 10^−7^	1.0 × 10^−4^–1.0 × 10^−4^	1.0 × 10^−4^ ± 2.0 × 10^−7^
Ni^2+^	3.0 × 10^−2^–5.0 × 10^−1^	2.0 × 10^−1^ ± 1.0 × 10^−2^	3.0 × 10^−2^–5.0 × 10^−2^	4.0 × 10^−2^ ± 3.0 × 10^−2^
Mn^2+^	6.0 × 10^−2^–5.0 × 10^−1^	3.0 × 10^−1^ ± 1.0 × 10^−2^	6.0 × 10^−3^–3.0 × 10^−2^	1.0 × 10^−3^ ± 2.0 × 10^−4^
Mn^3+^	2.0 × 10^−23^–1.94 × 10^−22^	1.0 × 10^−22^ ± 5.0 × 10^−23^	3.0 × 10^−22^–6.0 × 10^−22^	5.0 × 10^−22^ ± 1.0 × 10^−22^
Cr^3+^	7.0 × 10^−7^–7.12 × 10^−6^	4.0 × 10^−6^ ± 2.0 × 10^−8^	8.0 × 10^−6^–1.0 × 10^−5^	9.0 × 10^−6^ ± 1.0 × 10^−6^
Cr^6+^	8.0 × 10^−12^–8.06 × 10^−11^	4.0 × 10^−11^ ± 2.0 × 10^−10^	8.0 × 10^−11^–1.0 × 10^−10^	9.0 × 10^−11^ ± 1.0 × 10^−11^
Cu^1+^	3.0 × 10^−4^–5.63 × 10^−2^	9.0 × 10^−3^ ± 1.0 × 10^−2^	3.0 × 10^−3^–6.0 × 10^−2^	4.0 × 10^−4^ ± 1.0 × 10^−2^
Cu^2+^	7.0 × 10^−3^–1.30 × 10^−2^	2.0 × 10^−1^ ± 2.0 × 10^−3^	8.0 × 10^−4^–6.0 × 10^−4^	1.0 × 10^−3^ ± 3.0 × 10^−4^
Cd^2+^	1.0 × 10^−2^–3.0 × 10^−1^	7.0 × 10^−2^ ± 6.0 × 10^−3^	3.0 × 10^−4^–2.0 × 10^−4^	4.0 × 10^−4^ ± 2.0 × 10^−4^
Pb^2+^	8.0 × 10^−3^–2.0 × 10^−2^	5.0 × 10^−2^ ± 1.0 × 10^−2^	2.0 × 10^−4^–1.0 × 10^−4^	4.0 × 10^−3^ ± 8.0 × 10^−2^
Pb^4+^	1.0 × 10^−2^–1.0 × 10^−1^	2.0 × 10^−1^ ± 2.0 × 10^−3^	2.0 × 10^−2^–2.0 × 10^−1^	5 × 10^−2^ ± 6.0 × 10^−3^
Co^2+^	7.0 × 10^−3^–2.0 × 10^−2^	6.0 × 10^−2^ ± 4.0 × 10^−2^	2.0 × 10^−4^–4.0 × 10^−3^	3.0 × 10^−3^ ± 6.0 × 10^−2^
Co^3+^	9.0 × 10^−32^–2.3 × 10^−30^	8.0 × 10^−31^ ± 6.0 × 10^−31^	3.0 × 10^−30^–6.0 × 10^−29^	5.0 × 10^−30^ ± 1.0 × 10^−30^
Fe^2+^	6.0 × 10^−2^–2.0 × 10^−1^	3.0 × 10^−1^ ± 2.0 × 10^−3^	2.0 × 10^−3^–1.5 × 10^−3^	4.0 × 10^−3^ ± 1.0 × 10^−4^
Fe^3+^	7.0 × 10^−11^–1.05 × 10^−9^	4.0 × 10^−10^ ± 3.0 × 10^−10^	2.0 × 10^−9^–3.0 × 10^−9^	2.0 × 10^−9^ ± 3.0 × 10^−10^
Zn^2+^	6.0 × 10^−2^–2.0 × 10^−1^	2.0 × 10^−1^ ± 1.0 × 10^−1^	4 × 10^−3^–1.0 × 10^−2^	8.0 × 10^-^ ± 2.0 × 10^−4^

**Table 3:** Mineral phases of groundwater in comparison with mine water in the Adenzai flood plain region.

Statistic	Groundwater (*n* = 50)		Mines Water (*n* = 7)		Formula
	Range	Mean ± SD	Range	Mean ± SD	
Bunsenite	−3.06–1.84	−2.3 ± 0.308	−2.2–2.0	−2.09 ± 0.07	NiO
Ni (OH)_2_	−3.35–2.12	−2.6 ± 0.308	−2.5–2.28	−2.37 ± 0.06	Ni (OH)_2_
Trevorite	15.43–18.16	17.0 ± 0.803	18.3–18.6	18.5 ± 0.11	NiFe^3+^_2_O_4_
Birnessite	−60.2–52.9	−56.0 ± 2.038	−53.0–50.4	−51.4 ± 0.71	MnO_2_
Bixbyite	−8.79–6.97	−7.7 ± 0.509	−6.9–6.33	−6.59 ± 0.18	Mn_2_O_3_
Hausmannite	−10.2–7.47	−8.5 ± 0.763	−7.4–6.51	−6.91 ± 0.27	Mn_3_O_4_
Manganite	−4.71–3.8	−4.2 ± 0.254	−3.8–3.48	−3.62 ± 0.09	MnOOH
Manganosite	−8.22–7.31	−7.7 ± 0.255	−7.3–6.99	−7.13 ± 0.1	MnO
Pyrolusite	−9.87–8.96	−9.3 ± 0.255	−8.9–8.64	−8.77 ± 0.09	MnO_2_
Todorokite	−48.2–14.8	−44 ± 4.52	−42–39.6	−40.5 ± 0.63	(Mn^2+^, Ca, Na, K)(Mn^4+^, Mn^2+^, Mg)_6_O_12_·3H_2_O
Chromite	18.1–20.75	19.8 ± 0.595	20.8–21.2	21.0 ± 0.11	FeCr_2_O_4_
Eskolaite	15.63–17.58	17 ± 0.365	17.4–17.7	17.5 ± 0.1	Cr_2_O_3_
CuCr_2_O_4_	16.48–19.21	18.3 ± 0.616	19.6–20.0	19.8 ± 0.11	CuCr_2_O_4_
Cuprite	2.86–7.33	5.38 ± 0.943	6.87–7.4	7.06 ± 0.18	Cu_2_O
Delafossite	14.59–17.75	16.5 ± 0.674	17.8–18.1	17.9 ± 0.1	CuFeO_2_
Ferrite-Cu	13.82–17.89	16.3 ± 0.957	18.3–18.6	18.4 ± 0.13	CuFe_2_O_4_
Tenorite	1.09–3.33	2.34 ± 0.463	3.09–3.36	3.19 ± 0.09	CuO
Monteponite	−6.2–4.72	−5.5 ± 0.363	−4.8–4.63	−4.69 ± 0.05	CdO
Crocoite	−6.27–4.59	−5.5 ± 0.55	−4.5–4.16	−4.29 ± 0.13	PbCrO_4_
Litharge	−3.97–2.53	−3.3 ± 0.471	−2.5–2.16	−2.3 ± 0.1	PbO
Massicot	−4.01–2.18	−3.5 ± 0.491	−2.7–2.34	−2.48 ± 0.1	PbO
Minium	−26–21.1	−23.0 ± 1.426	−21–20	−20.4 ± 0.31	Pb_3_O_4_
Plattnerite	−18.4–17.1	−18 ± 0.465	−17.0–16.7	−16.9 ± 0.1	PbO_2_
Spinel-Co	−11.4–7.15	−8.9 ± 1.071	−7.3–6.39	−6.75 ± 0.28	Co-MgAl_2_O_4_
Goethite	−9.84–8.25	7.42 ± 2.512	8.35–8.5	8.41 ± 0.05	FeO(OH)
Hematite	15.22–17.49	16.5 ± 0.67	17.6–17.9	17.8 ± 0.1	Fe_2_O_3_
Magnetite	14.54–17.94	16.5 ± 1.006	−19.0–18.5	9.16 ± 17.0	Fe_3_O_4_
Wustite	−3.47–2.39	−2.8 ± 0.317	−2.3–2.21	−2.26 ± 0.03	FeO
Ferrite-Zn	13.89–16.23	15.1 ± 0.696	16.7–17.0	16.8 ± 0.09	ZnFe_2_O_4_
Zincite	−1.27–0.52	−1.0 ± 0.166	−0.7–0.42	−0.52 ± 0.1	ZnO
ZnCr_2_O_4_	25.74–27.61	26.9 ± 0.393	27.7–28.2	27.9 ± 0.17	ZnCr_2_O_4_
T. Hydrogen	111.0–111.2	111.0 ± 0.004	111.0–111.5	111.0 ± 0.04	
T. Oxygen	55.53–55.83	55.5 ± 0.044	55.5–55.6	55.5 ± 0.02	
Ionic strength	0.001–0.007	0.005 ± 0.001	0.01–0.02	0.02 ± 0.01	

**Table 4:** Describe the non-cancer risk and the cancer risk of PHE consumption through oral ingestion of groundwater from the Adenzai flood plain region of Pakistan.

	**Non-Cancer Risk in Children**		**Non-Cancer Risk in Male**		**Non-Cancer Risk in Female**	
	**Range**	**Mean ± SD**	**Range**	**Mean ± SD**	**Range**	**Mean ± SD**
Mn	0.044–1.25	0.3 ± 0.2647	0.018–0.52	0.125 ± 0.11	0.019–0.55	0.13 ± 0.115
Cu	0.03–6.76	1.301 ± 1.5718	0.012–2.81	0.552 ± 0.673	0.013–2.94	0.58 ± 0.706
Co	0.102–9.63	2.178 ± 2.2921	0.042–4.0	0.905 ± 0.952	0.044–4.19	0.95 ± 0.998
Fe	2 × 10^−5^–0.002	1 × 10^−4^ ± 0.0001	7 × 10^−6^–0.001	6 × 10^−5^ ± 5 × 10^−5^	8 × 10^−6^–0.005	0.002 ± 5 × 10^−5^
Zn	0.041–0.3	0.106 ± 0.0666	0.017–0.14	0.044 ± 0.029	0.018–0.15	0.05 ± 0.03
THI	0.217–17.9	3.885 ± 4.1953	0.09–7.47	1.626 ± 1.764	0.094–7.83	1.7 ± 1.85
	**Cancer Risk in Children**		**Cancer Risk in Male**		**Cancer Risk in Female**	
Ni	1 × 10^−7^–0.05	0.024 ± 0.0149	0.001–0.02	0.011 ± 0.006	0.001–0.02	0.01 ± 0.006
Cr	6 × 10^−4^–0.02	0.005 ± 0.0039	2 × 10^−4^–0.01	0.002 ± 0.001	2 × 10^−4^–0.01	2 × 10^−1^ ± 0.002
Cd	0.017–0.8	0.176 ± 0.2146	0.007–0.33	0.074 ± 0.09	0.007–0.35	0.08 ± 0.095
Pb	0.009–0.55	0.093 ± 0.1297	0.004–0.23	0.04 ± 0.054	0.004–0.24	0.04 ± 0.057
THI	0.027–1.42	0.298 ± 0.3631	0.013–0.59	0.126 ± 0.152	0.013–0.62	0.13 ± 0.16

**Table 5:** Nemerow’s pollution indexing representing the water quality status of three hydrological environments in the groundwater system compared with mine water.

Statistic	Shallow Groundwater (*n* = 24)		Mid-Depth Water (*n* = 14)		Deep Groundwater (*n* = 12)		Mines Water (*n* = 7)	
	Range	Mean ± SD	Range	Mean ± SD	Range	Mean ± SD	Range	Mean ± SD
pH	0.85–0.98	0.9 ± 0.03	0.824–0.95	0.87 ± 0.04	0.847–0.95	0.89 ± 0.03	0.89–0.96	0.93 ± 0.02
EC	0.53–3.22	1.85 ± 0.66	0.833–2.58	1.69 ± 0.53	1.174–2.8	1.82 ± 0.54	4.13–4.63	4.5 ± 0.17
TDS	0.21–0.8	0.46 ± 0.16	0.21–0.64	0.41 ± 0.13	0.3–0.68	0.45 ± 0.13	1.05–1.28	1.13 ± 0.1
Ca	0.27–1.0	0.41 ± 0.15	0.34–0.85	0.55 ± 0.16	0.28–1.2	0.67 ± 0.31	0.2–0.29	0.25 ± 0.03
Mg	0.3–0.66	0.51 ± 0.09	0.36–0.74	0.59 ± 0.12	0.36–0.9	0.57 ± 0.17	0.2–0.42	0.3 ± 0.07
K	0.38–1.58	0.75 ± 0.25	0.37–0.9	0.69 ± 0.21	0.075–0.9	0.51 ± 0.29	0.15–1.0	0.48 ± 0.32
Na	0.28–1.75	0.81 ± 0.32	0.22–0.85	0.47 ± 0.21	0.11–0.75	0.43 ± 0.22	1.68–2.05	1.84 ± 0.13
HCO_3_	0.7–2.83	1.02 ± 0.41	0.6.0–1.12	0.86 ± 0.18	0.63–1.1	0.88 ± 0.15	2.03–2.27	2.17 ± 0.09
Cl	0.32–0.6	0.46 ± 0.08	0.22–0.58	0.38 ± 0.08	0.32–0.54	0.42 ± 0.07	0.32–0.48	0.38 ± 0.06
SO_4_	0.23–0.48	0.33 ± 0.07	0.17–0.47	0.3 ± 0.07	0.16–0.3	0.26 ± 0.05	0.65–0.7	0.67 ± 0.02
Ni	0.02–0.18	0.08 ± 0.05	0.01–0.18	0.07 ± 0.05	0.01–0.13	0.06 ± 0.04	0.11–0.17	0.14 ± 0.03
Mn	0.11–1.0	0.62 ± 0.26	0.16–1.0	0.49 ± 0.31	0.16–0.7	0.45 ± 0.19	1.3–3.16	2.29 ± 0.65
Cr	0.32–0.6	0.46 ± 0.08	0.22–0.58	0.38 ± 0.08	0.32–0.54	0.42 ± 0.07	0.32–0.48	0.38 ± 0.06
Cu	0.02–0.95	0.24 ± 0.23	0.01–0.23	0.07 ± 0.06	0.01–0.12	0.06 ± 0.04	0.6–1.13	0.75 ± 0.2
Cd	0.2–6.2	1.49 ± 1.51	0.22–4.6	1.4 ± 1.32	0.2–1.2	0.72 ± 0.29	7.0–9.6	8.2 ± 1.01
Pb	1.0–20.0	6.88 ± 6.04	1.0–16.1	7.09 ± 5.61	1.0–4.0	1.75 ± 1.06	25.0–58.0	42.6 ± 12.3
Co	0.14–6.0	1.78 ± 1.48	0.75–5.75	2.29 ± 1.39	0.75–5.25	1.88 ± 1.36	6.0–13.0	10.0 ± 2.45
Fe	0.77–4.47	2.88 ± 1.25	0.8–5.2	2.35 ± 1.69	0.37–1.03	0.78 ± 0.22	7–9.5.0	8.4 ± 0.97
Zn	0.04–0.22	0.08 ± 0.04	0.04–0.12	0.07 ± 0.02	0.04–0.12	0.08 ± 0.02	0.15–0.3	0.23 ± 0.05

**Table 6:** Principal component analysis multilinear regression results of the groundwater system in comparison with mine water in the Adenzai flood plain region, Pakistan. Bold values are different from others.

	Groundwater (*n* = 50)			Mines Water (*n* = 7)		
	F1	F2	F3	F1	F2	F3
pH	**0.658**	−0.273	−0.359	**0.579**	0.577	**0.700**
EC	**0.561**	**−0.623**	0.071	**0.643**	−0.436	0.192
Temp	0.367	0.062	−0.152	0.175	**0.764**	**0.602**
Depth	**−0.652**	**−0.521**	−0.140	−0.409	**−0.694**	0.297
TDS	**0.585**	**−0.613**	0.096	**0.594**	−0.032	−0.031
Ca	**−0.648**	0.090	−0.218	**−0.540**	**−0.696**	0.049
Mg	**−0.512**	0.098	0.230	**−0.588**	**−0.535**	0.402
K	0.390	0.168	0.260	**0.932**	−0.037	−0.210
Na	**0.883**	−0.214	0.016	**0.733**	**0.579**	−0.332
HCO_3_	**0.623**	−0.354	−0.392	0.452	**0.765**	**−0.562**
Cl	0.288	−0.048	**0.714**	**0.921**	0.272	−0.010
SO_4_	**0.762**	−0.012	0.069	**0.842**	−0.122	**−0.500**
Ni	0.107	**0.547**	**−0.527**	**0.923**	−0.249	−0.143
Mn	**0.523**	0.358	0.203	**0.892**	−0.345	0.265
Cr	0.205	**0.734**	−0.254	**0.633**	−0.098	**0.718**
Cu	0.291	**0.515**	0.077	−0.296	**0.498**	−0.040
Cd	**0.541**	0.220	−0.054	0.418	**0.464**	**0.751**
Pb	**0.520**	0.408	−0.210	**0.642**	**−0.735**	0.193
Co	−0.210	−0.069	**0.586**	**0.714**	**−0.609**	0.023
Fe	0.477	**0.671**	0.285	**0.873**	−0.255	−0.124
Zn	0.101	0.010	0.037	−0.052	**0.871**	0.113
Eigenvalue	4.952	2.985	1.826	6.137	3.285	2.012
Variability (%)	40.285	23.526	16.412	43.482	25.936	16.034
Cumulative %	40.285	63.811	80.223	43.482	69.418	85.452

## Data Availability

Research data can be obtained from the corresponding author through email.

## Supplementary Materials

The following supporting information can be downloaded at: https://www.mdpi.com/article/10.3390/ijerph19116472/s1, Figure S1: Reveals concentration profile of Cr, vs. Ni, Mn, Cu, Cd, Pb, Co, Fe, and Zn of groundwater in the Adenzai, Pakistan. Figure S2: The concentrations plot of Cd vs. pH, Ni, Mn, Cr, Cu, Pb, Co, Fe, and Zn in the groundwater Adenzai, Pakistan. Figure S3: The concentration profile of Pb vs pH, Ni, Mn, Cr, Cu, Cd, Co, Fe, and Zn of groundwater in the Adenzai, Pakistan. Figure S4: Shows northing plots against pH, Ni, Mn, Cr, Cu, Cd, Pb, Co, and Zn of groundwater in the Adenzai region, Pakistan. Figure S5: Profile plot of overall parameters of groundwater in the Adenzai, flood plain area Pakistan. Table S1: Pearson correlation of potentially harmful elements in groundwater of Adenzai flood plain region of Pakistan. Table S2: Represent the pollution index of potentially harmful elements of groundwater in the Adenzai flood plain region of Pakistan.
