# Supplementary material for: Geochemical Modeling Source Provenance, Public Health Exposure, and Evaluating Potentially Harmful Elements in Groundwater: Statistical and Human Health Risk Assessment (HHRA)
Source: Int J Environ Res Public Health. 2022 May 26;19(11):6472. doi: 10.3390/ijerph19116472 (PMC9180908; doi:10.3390/ijerph19116472)
Supplement: Supplementary file 1 [file ijerph-19-06472-s001.zip › ijerph-1657882-supplementary.pdf]

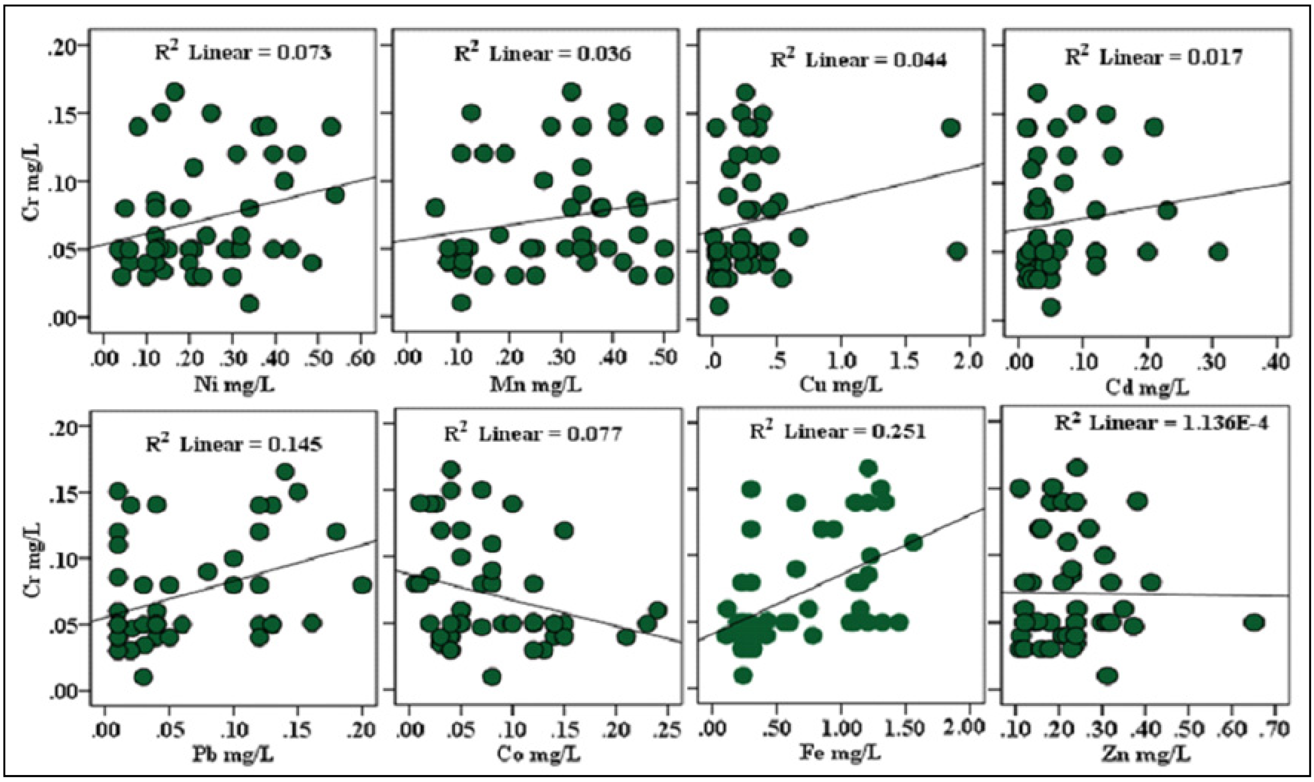

Figure S1. Reveals concentration profile of Cr, vs. Ni, Mn, Cu, Cd, Pb, Co, Fe, and Zn of groundwater in the Adenzai, Pakistan.

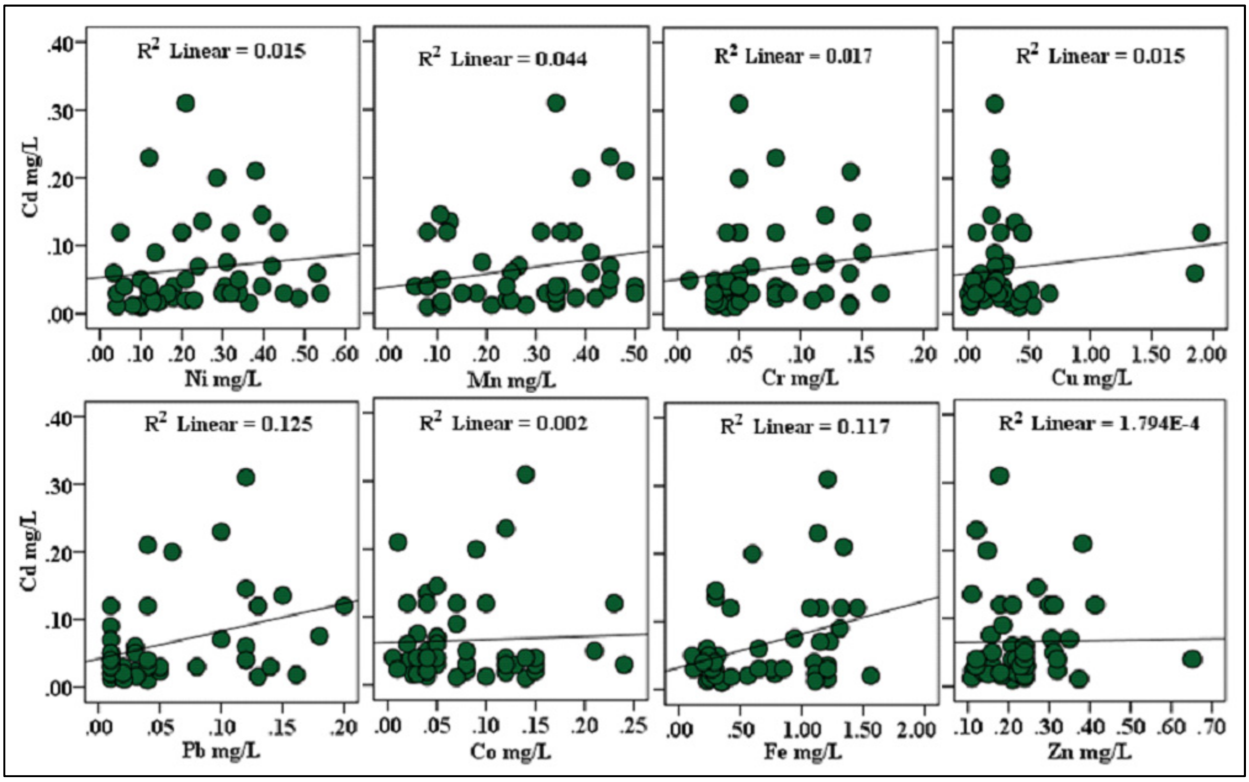

Figure S2. The concentrations plot of Cd vs. pH, Ni, Mn, Cr, Cu, Pb, Co, Fe, and Zn in the groundwater Adenzai, Pakistan.

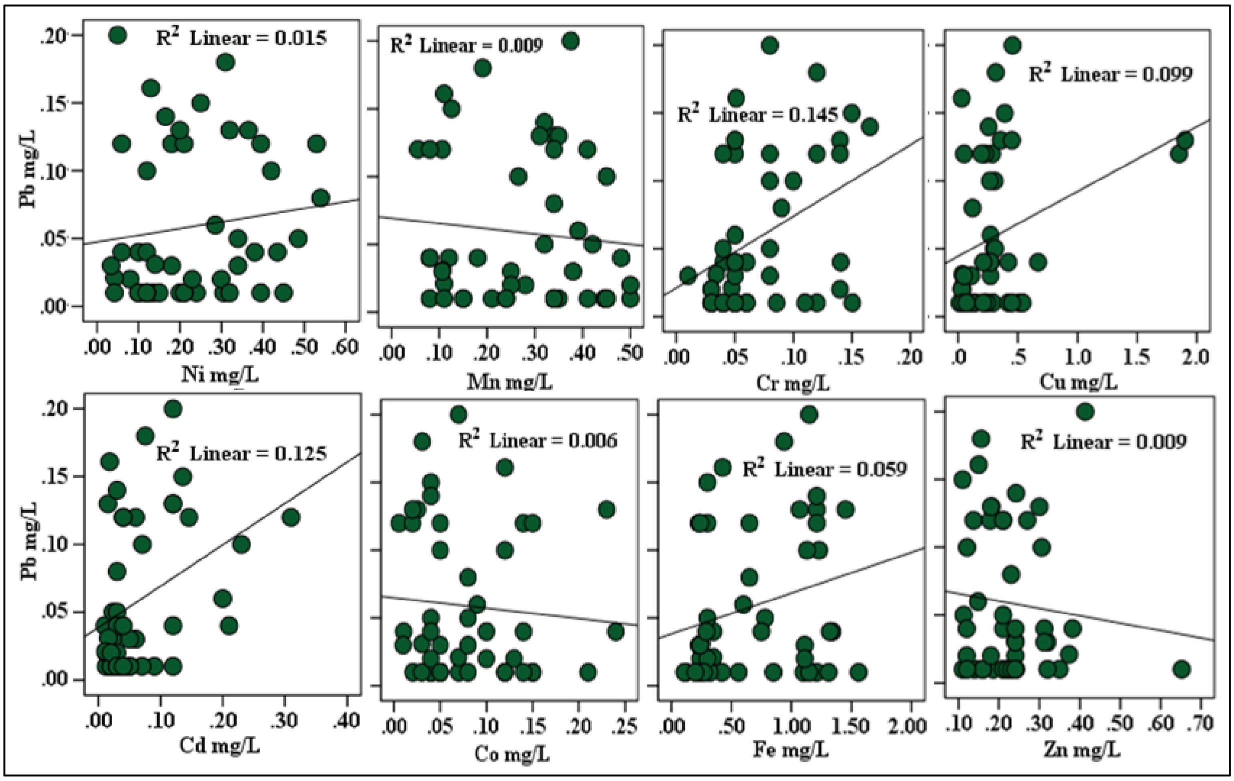

Figure S3. The concentration profile of Pb vs pH, Ni, Mn, Cr, Cu, Cd, Co, Fe, and Zn of groundwater in the Adenzai, Pakistan.

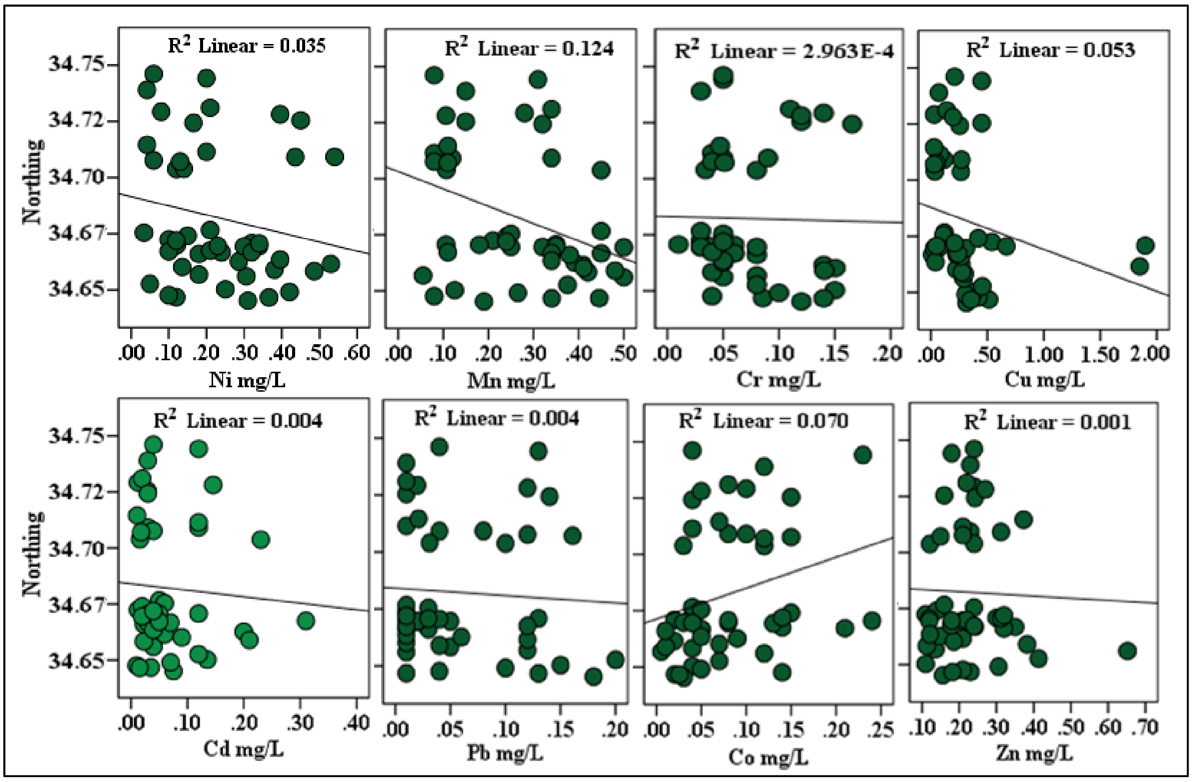

Figure S4. Shows northing plots against pH, Ni, Mn, Cr, Cu, Cd, Pb, Co, and Zn of groundwater in the Adenzai region, Pakistan.

Table S1. Pearson correlation of potentially harmful elements in groundwater of Adenzai flood plain region of Pakistan.

Variables	pH	EC	Temp	Depth	TDS	Ca	Mg	K	Na	HCO_3	Cl	SO_4	Ni	Mn	Cr	Cu	Cd	Pb	Co	Fe	Zn
pH	**1.00**																				
EC	**0.70**	**1.00**																			
Temp	0.40	0.25	**1.00**																		
Depth	-0.32	-0.30	-0.19	**1.00**																	
TDS	**0.72**	**0.98**	0.25	-0.31	**1.00**																
Ca	-0.30	-0.47	-0.23	0.49	-0.48	**1.00**															
Mg	-0.37	-0.56	-0.08	0.36	-0.56	**0.72**	**1.00**														
K	0.04	-0.16	0.05	-0.25	-0.10	-0.17	0.14	**1.00**													
Na	**0.70**	**0.83**	0.26	-0.56	**0.84**	**-0.71**	**-0.76**	0.54	**1.00**												
HCO_3	**0.72**	**0.81**	0.24	-0.41	**0.81**	**-0.70**	**-0.72**	-0.10	**0.91**	**1.00**											
Cl	0.05	-0.06	0.03	-0.14	-0.02	-0.16	0.19	0.16	0.04	-0.20	**1.00**										
SO_4	**0.73**	**0.83**	0.22	-0.50	**0.84**	-0.51	-0.53	-0.05	**0.90**	**0.84**	-0.09	**1.00**									
Ni	0.28	0.34	0.27	-0.28	0.36	-0.07	-0.31	-0.21	0.31	0.33	-0.16	0.35	**1.00**								
Mn	0.37	**0.75**	0.25	-0.40	**0.79**	-0.33	-0.51	-0.08	**0.72**	0.66	-0.02	**0.82**	0.50	**1.00**							
Cr	0.28	**0.71**	0.23	-0.51	0.64	-0.35	-0.52	-0.04	0.67	0.65	-0.24	0.75	0.50	**0.80**	**1.00**						
Cu	0.31	0.41	0.14	-0.49	0.61	-0.29	-0.44	-0.20	0.67	0.63	0.05	**0.70**	0.45	0.66	0.67	**1.00**					
Cd	0.41	**0.79**	0.36	-0.43	**0.80**	-0.42	-0.55	-0.19	**0.77**	**0.72**	-0.16	**0.83**	0.42	**0.82**	**0.78**	**0.70**	**1.00**				
Pb	0.35	0.50	0.13	-0.43	**0.77**	-0.38	-0.49	-0.09	**0.73**	**0.74**	-0.18	0.52	0.45	**0.84**	**0.83**	0.69	**0.84**	**1.00**			
Co	0.20	0.48	0.08	-0.29	**0.76**	-0.31	-0.48	-0.13	0.65	0.63	-0.06	**0.74**	0.31	**0.80**	0.67	0.60	**0.78**	**0.82**	**1.00**		
Fe	0.32	0.45	0.24	**-0.63**	0.69	-0.46	-0.48	0.01	**0.74**	0.64	-0.02	0.50	0.46	**0.85**	0.84	0.69	**0.82**	**0.80**	**0.74**	**1.00**	
Zn	0.35	0.62	0.28	-0.27	**0.72**	-0.33	-0.51	-0.23	**0.70**	0.68	-0.13	**0.76**	0.35	**0.72**	0.67	0.63	**0.74**	0.66	0.65	**0.73**	**1.00**

Note: Values in bold are different from 0 with a significance level alpha=0.05.

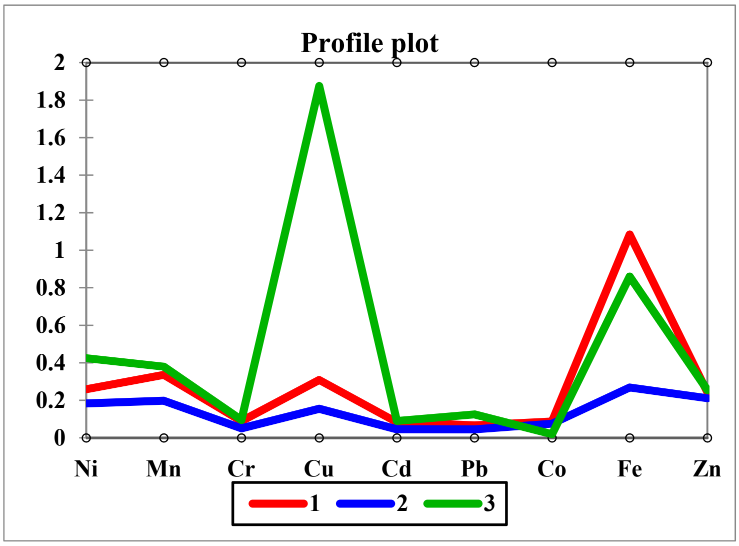

Figure S5. Profile plot of overall parameters of groundwater in the Adenzai, flood plain area Pakistan.

Table S2. Represent the pollution index of potentially harmful elements of groundwater in the Adenzai flood plain region of Pakistan.

Comp	Mean	Background Value	CF
Ni	0.23	0.20	1.15
Mn	0.27	0.20	1.35
Cr	0.07	0.04	1.79
Cu	0.30	0.11	2.80
Cd	0.07	0.04	1.75
Pb	0.06	0.02	3.36
Co	0.12	0.02	2.11
Fe	0.93	0.3	2.09
Zn	0.23	0.24	0.97
